# A Two-Photon Zn(II) Complex Photosensitizer with pH/Viscosity Dual Response for Enhanced Tumor Therapy

**DOI:** 10.3390/molecules30112430

**Published:** 2025-05-31

**Authors:** Yu Zhang, Shao-Qi Guan, Ya-Ping Wang, Mei Pan

**Affiliations:** MOE Laboratory of Bioinorganic and Synthetic Chemistry, Lehn Institute of Functional Materials, IGCME, GBRCE for Functional Molecular Engineering, School of Chemistry, Sun Yat-sen University, Guangzhou 510006, China; zy6535337@163.com (Y.Z.); guanshq6@mail2.sysu.edu.cn (S.-Q.G.); yapingwang0393@163.com (Y.-P.W.)

**Keywords:** Zn(II) complex, TP fluorescence, dual monitoring, ROS, PDT

## Abstract

In recent years, an increasing number of studies have shown that novel metal complexes with bio-imaging capabilities could enhance precision oncology, particularly through optimized photosensitizer (PS) design for subcellular organelle targeting photodynamic therapy (PDT). Based on this, we successfully developed a two-photon (TP) fluorescent Zn(II) complex, LIFM–ZY–3, characterized by the specifical targeting capability of lysosome. This complex was capable of monitoring dual changes in pH and viscosity. Additionally, our findings indicated that the complex could generate multiple reactive oxygen species (ROS), including singlet oxygen (^1^O_2_), hydroxyl radicals (•OH), and superoxide anion radicals (O_2_^−^) under white light irradiation in vivo and in vitro. These findings underscored the remarkable versatility of LIFM–ZY–3 as an advanced multifunctional PS for both microenvironment monitoring and tumor therapy.

## 1. Introduction

Recent advancements in tumor therapy and pathogenesis research have highlighted targeted subcellular organelle therapy for its ability to enhance therapeutic specificity [1,2,3]. Lysosomes are the “digestive system” within cells, responsible for degrading and recycling intracellular and extracellular waste and damaged molecules [4]. In tumor cells, lysosomal function is often abnormal, making them a potential target for cancer therapy [5]. In recent years, significant progress has been achieved in the application of lysosome-targeted therapy for tumor treatment [6]. A notable advancement is lysosome-targeted photodynamic therapy (LTPDT), which utilizes photosensitizers (PS) to specifically localize within lysosomes [7]. Upon light activation, these photosensitizers generate reactive oxygen species (ROS), leading to lysosomal membrane permeabilization and subsequent induction of cell death. This approach enhances the precision and efficacy of tumor treatment by selectively targeting lysosomal pathways in cancer cells.

The tumor microenvironment (TME) is acidic (pH 6.0–7.0), with lysosomal pH even lower (4.5–5.5) [8]. Acidic conditions influence PS properties, altering absorption spectra and reducing ROS generation efficiency [9]. Low pH also enhances lysosomal membrane sensitivity to ROS, promoting lysosomal membrane permeabilization (LMP), which triggers hydrolytic enzyme release, leading to apoptosis or necrosis and improving PDT efficacy. Furthermore, ROS stabilization under acidic conditions may contribute to the enhanced activity of pH-sensitive PSs [10]. Elevated viscosity, another key TME feature [11], synergizes with low pH to enhance tumor invasion and metastasis [12]. Since pH and viscosity are interconnected yet independent, dual monitoring is essential for accurate tumor diagnosis and staging [13]. Developing novel fluorescent probes and multimodal imaging technologies for dual monitoring can advance precision oncology, particularly in optimizing PS design for subcellular organelle-targeted tumor therapy.

Fluorescent Zn(II) complexes exhibit significant potential as PSs in PDT and biological imaging [14] due to their unique photophysical properties, excellent biocompatibility, and tunable photosensitizing performance, making them ideal candidates for tumor treatment, antibacterial therapy, and photodynamic diagnosis. The integration of metal Zn(II) complexes with two-photon (TP) property can optimize their photophysical properties with fluorescence modulation capabilities in deeper tissue penetration and high spatial resolution, and PDT efficacy [15]. The naphthalene imide derivatives with TP property, [16] as multifunctional ligands, improve the optical properties, TME responsiveness, and targeted therapeutic effects of Zn(II) complexes. By combining PDT with real-time TME sensing, Zn(II) complexes open new avenues for advancing cancer treatment strategies.

Building on these foundations, we designed and synthesized a lysosome-targeted Zn(II) complex by integrating naphthalene imide derivatives with the metal zinc ion. The complex had pH and viscosity dual-responsive TP emission, and it exhibited superior LTPDT efficacy compared to the ligand alone. Notably, TP lysosome-targeted PS combined with Zn(II) complexes for detecting dual pH/viscosity remained rare, positioning our work as a significant contribution to the field. We anticipated that Zn(II) complexes could be employed for self-reporting dynamics PDT by taking the exceptional tumor micro-environment. Through further optimization and clinical translation, Zn(II) complexes were poised to become essential tools in precision medicine, offering new solutions for cancer treatment.

## 2. Results and Discussion

### 2.1. Optical Properties

**Scheme 1:**
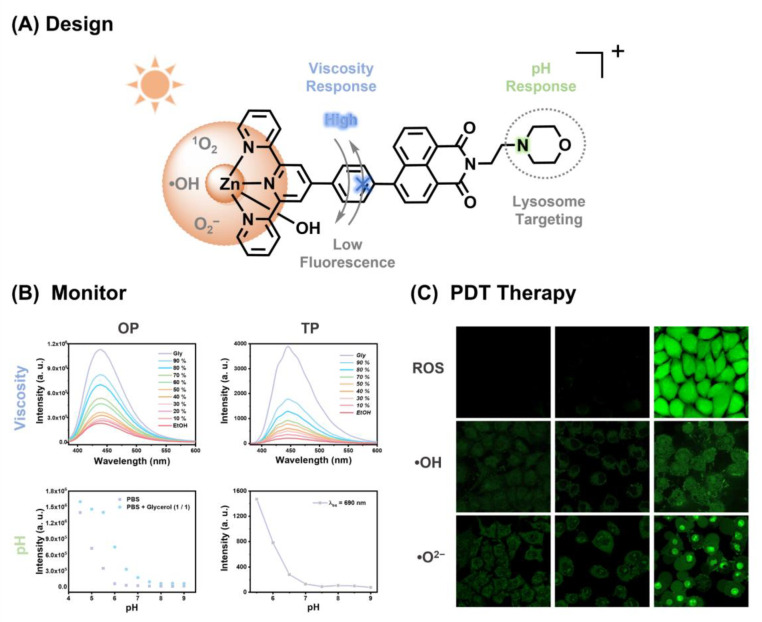
(**A**) The design of tetrahedrally–coordinated zinc(II) complex LIFM–ZY–3. (**B**) The complex LIFM–ZY–3 can monitor the viscosity and pH dual changes in vitro. (**C**) The PDT efficacy of LIFM–ZY–3 was evaluated at the cellular level.

The lysosome-targeted TP fluorescent Zn(II) complex, LIFM–ZY–3, was designed to exhibit dual responsiveness of pH and viscosity. Also, it was constructed by linking a naphthalene imide derivative with a zinc ion via coordination bonding, as illustrated in Scheme 1. The viscosity response mechanism of LIFM–ZY–3 was governed by the twisted intramolecular charge transfer (TICT) mechanism with a rotatable benzene ring [17]. In contrast, the pH response mechanism of LIFM–ZY–3 relied on the reversible protonation and deprotonation of the morpholine moiety, which modulates the N lone pair of electrons on the ligand [18]. In addition, the morpholine group had superior targeting lysosome property, [19], and it was widely utilized in bio-imaging applications due to its versatility in biology. The synthetic routes of ligand L_3_ and complex LIFM–ZY–3 were detailed in Scheme S1, and the structures of L_3_ and LIFM–ZY–3 were fully characterized using ^1^H NMR, ^13^C NMR, and HRMS in Figures S1–S7.

**Figure 1:**
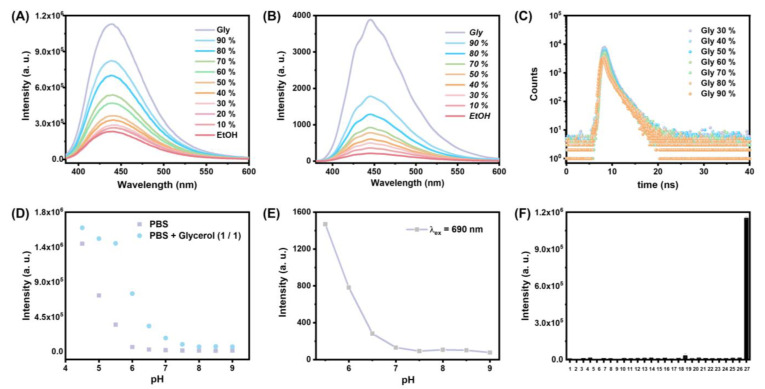
(**A**) The fluorescence emission spectrum of LIFM–ZY–3 (5 μM) in the changed viscosity media of the EtOH/glycerol (*v*/*v*) mixtures. (**B**) The TP induced emission spectrum of LIFM–ZY–3 (10 μM) in the changed viscosity media of the EtOH/glycerol (*v*/*v*) mixtures at 690 nm. (**C**) The time–resolved emission decays of LIFM–ZY–3 (10 μM) at different viscosities in EtOH/glycerol (*v*/*v*) mixtures. (**D**) The pH–responsive fluorescence intensity changes in the complex LIFM–ZY–3 (5 μM) in different viscosity systems. (**E**) The TP fluorescence intensity of LIFM–ZY–3 (10 μM) at changed pH environments at 460 nm. (**F**) The changes in fluorescence intensity of LIFM–ZY–3 (5 μM) treated with different interfering substances 1–27 (Fe^3+^, Co^2+^, Zn^2+^, Ca^2+^, Cu^2+^, Mg^2+^, Mn^2+^, Cu^+^, K^+^, Na^+^, Cysteine, GSH, H_2_O_2_, OCl^−^, CH_3_COO^−^, NO_2_^−^, NO_3_^−^, HS^−^, HSO_3_^−^, HSO_4_^−^, SO_4_^2−^, F^−^, Cl^−^, Br^−^, I^−^, and glycerol) at 450 nm.

We initially investigated the optical properties of the complex LIFM–ZY–3. All spectroscopic measurements were performed in a mixed ethanol–glycerol solvent system to simulate and establish an in vitro viscous environment. As shown in Figure S8A, the absorption spectra of LIFM–ZY–3 in various solvents exhibited characteristic absorption peaks at 350 nm. Compared to other solvents, the fluorescence intensity of LIFM–ZY–3 displayed apparent enhancement in glycerol in Figure S8B, indicating that the complex could detect viscosity changes. Subsequently, we further analyzed the fluorescence changes in LIFM–ZY–3 in solutions with varying viscosities. As depicted in Figure 1A, the fluorescence intensity of LIFM–ZY–3 exhibited a significant enhancement, increasing up to 5-fold, in response to elevated viscosity. Also, there was a strong linear correlation between the log of viscosity changes and the log of fluorescence intensity at 438 nm in Figure S9. These results demonstrated that the complex LIFM–ZY–3 was capable of quantitatively detecting and measuring changes in viscosity.

Considering the broad biological applicability of Zn(II) complexes in responding to TME and cell imaging, we further explored the TP properties of LIFM–ZY–3 to gain deeper insights into its potential for high-resolution, deep-tissue imaging and its interactions with complicated cellular environments. As shown in Figure S10A,B, LIFM–ZY–3 exhibited a maximum TP absorption cross-section of 75 GM at 670 nm. Furthermore, a linear relationship was observed between the log of the emitted fluorescence intensity and the log of the excitation power, with a slope of 2 in Figure S10C,D, confirming the TP fluorescence characteristics of Zn(II) complex LIFM–ZY–3. Additionally, the TP fluorescence intensity of LIFM–ZY–3 exhibited a 17-fold increase in response to elevated viscosity in Figure 1B and Figure S11. It demonstrated that LIFM–ZY–3 is capable of achieving both OP and highly efficient TP responses to viscosity changes, underscoring its significant potential for deep-tissue biological imaging applications. Consistent with previous studies, most viscosity-responsive fluorescent probes have been reported to exhibit changes in fluorescence lifetime in response to viscosity variations [20]. Then, we investigated the fluorescence lifetime changes in LIFM–ZY–3 at different viscous solvents. As shown in Figure 1C, the fluorescence lifetime of LIFM–ZY–3 showed a significant increase in response to elevated viscosity. Moreover, a strong linear correlation between the fluorescence lifetime of LIFM–ZY–3 and viscosity were observed in Figure S12, demonstrating the capability of LIFM–ZY–3 to qualitatively detect and quantitatively measure viscosity changes through changing fluorescence lifetime.

Given the acidic nature of TME in biological system, [21] we investigated the pH–dependent behavior of LIFM–ZY–3 in Figure 1D. The fluorescence intensity of LIFM–ZY–3 exhibited a significant drop with increasing pH in both low–viscosity (PBS) and high–viscosity (PBS/glycerol = 1/1) conditions. Subsequently, we further investigated the TP fluorescence intensity of LIFM–ZY–3 under varying pH conditions. The TP fluorescence intensity of LIFM–ZY–3 gradually decreased with increasing pH conditions in Figure S13 and Figure 1E. And the quantum yield (QY) of LIFM–ZY–3 has been measured at various pH levels in Figure S14A, the QY of LIFM–ZY–3 decreased with increasing pH conditions, and a linear relationship was observed between the QY and the different fractions of glycerol, with 0.95 in Figure S14B. Collectively, these results demonstrated that the complex LIFM–ZY–3 could effectively monitor pH and viscosity changes under both OP and TP excitation, highlighting its potential for TME-sensitive applications in biological systems.

To eliminate potential interference from solvent polarity on the viscosity response, we examined the fluorescence spectrum of LIFM–ZY–3 under varying polarities. In a tetrahydrofuran–water mixed system, the complex LIFM–ZY–3 displayed 50 nm red shifts with increasing polarity in Figure S15; however, fluorescence intensity of LIFM–ZY–3 was notably lower compared to that observed in a glycerol solvent. Subsequently, we evaluated the selectivity of LIFM–ZY–3 against various potential interfering substances, including 1–27 (Fe^3+^, Co^2+^, Zn^2+^, Ca^2+^, Cu2^+^, Mg^2+^, Mn^2+^, Cu^+^, K^+^, Na^+^, Cysteine, GSH, H_2_O_2_, OCl^−^, CH_3_COO^−^, NO_2_^−^, NO_3_^−^, HS^−^, HSO_3_^−^, HSO_4_^−^, SO_4_^2−^, F^−^, Cl^−^, Br^−^, I^−^, and glycerol). As shown in Figure 1F, the fluorescence intensity of LIFM–ZY–3 at 450 nm remained largely unchanged in the presence of these interfering substances, demonstrating minimal interference compared to its response in viscous environments. Additionally, we evaluated the photostability of LIFM–ZY–3 in Figure S16. The result demonstrated that its fluorescence intensity remained nearly constant under continuous irradiation for up to 8 h. These findings confirmed that LIFM–ZY–3 could specifically detect viscosity and pH changes with high stability, demonstrating its adaptability to complicated biological systems and underscoring its strong potential for biological applications.

### 2.2. Quantification of ROS

**Figure 2:**
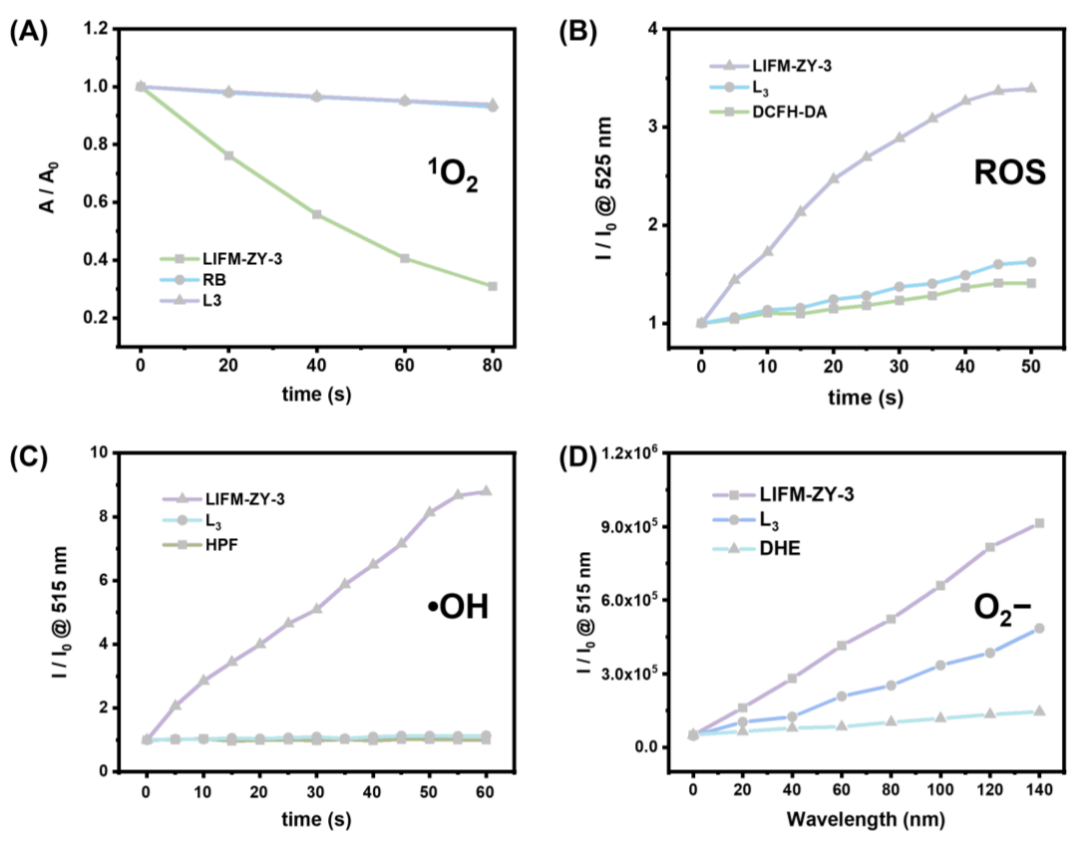
(**A**) Comparison of absorption intensity of complex LIFM–ZY–3, ligand L_3,_ and RB at different white light irradiation times and 380 nm wavelength in the same concentration. (**B**) At the same concentration, the fluorescence intensity of the complex LIFM–ZY–3, the ligand L_3_, and DCFH–DA at different white light irradiation times and 530 nm wavelength were compared. (**C**) At the same concentration, the fluorescence intensity of the complex LIFM–ZY–3, the ligand L_3_, and HPF at different white light irradiation times and 530 nm wavelength. (**D**) At the same concentration, the producing O_2_^−^ efficiency of the complex LIFM–ZY–3, the ligand L_3,_ and DHE at different white light irradiation times and 530 nm wavelength.

Considering the MLCT phenomenon observed in fluorescent Zn(II) complexes, [22] we further investigated the potential of LIFM–ZY–3 as a PS for PDT. Firstly, we evaluated the ability of LIFM–ZY–3 to generate singlet oxygen (^1^O_2_) using 9,10–anthracenediyl–bis(methylene)dimalonic acid (ABDA) [23] under white light irradiation at an intensity of 35 mW/cm^2^. As shown in Figure S17A, the maximal absorption of ABDA gradually decreased with increasing irradiation time, confirming the ^1^O_2_ generation capability of LIFM–ZY–3 increased steadily under 35 mW/cm^2^ white light irradiation. Additionally, we evaluated the ligand L_3_ under identical conditions and observed that it also generated ^1^O_2_, though with significantly lower efficiency compared to LIFM–ZY–3 in Figure S17B. For comparative analysis, we utilized rhodamine B (RhB), a conventional small molecule photosensitizer, as a reference to evaluate ROS generation efficiency [24]. As shown in Figure S17C, the maximum absorption of ABDA decreased with increasing irradiation time, which demonstrated that commercial RB could generate ^1^O_2_. In contrast to the complex LIFM–ZY–3, the ^1^O_2_ production efficiency of RB was significantly lower than that of LIFM–ZY–3 in Figure 2A, comparable to that of ligand L_3_ under identical conditions. These results demonstrated that LIFM–ZY–3 exhibited superior ^1^O_2_ generation efficiency compared to both ligand L_3_ and RB, emphasizing its potential as an effective photosensitizer for Type II photodynamic therapy.

To assess the potential of the complex LIFM–ZY–3 for biological applications, we adjusted the white light irradiance to a biologically relevant level of 20 mW/cm^2^ and re-evaluated the ^1^O_2_ production rate of LIFM–ZY–3. The result demonstrated that LIFM–ZY–3 still maintained high ^1^O_2_ production efficiency even at lower light irradiance in Figure S18, and there was a strong linear relationship (correlation coefficient = 0.999) between the maximum absorption of LIFM–ZY–3 and irradiation time. It indicated that LIFM–ZY–3 could stably and predictably generate ^1^O_2_ under light irradiation. Additionally, we employed a fluorescence–based method using 2′,7′–dichlorodihydrofluorescein diacetate (DCFH–DA) [25] to confirm the generation of ROS by complex LIFM–ZY–3 under light irradiation. As shown in Figure 2B and Figure S19, the enhancing fluorescence of DCFH–DA confirmed that the complex LIFM–ZY–3 could effectively produce ROS under white light irradiation.

Hydroxyphenyl fluorescein (HPF) selectively and dose-dependently reacts with hydroxyl radicals (•OH), leading to a corresponding increase in fluorescence intensity [26]. Taking advantage of this property, we evaluated the ability of the complex LIFM–ZY–3 to generate •OH under 20 mW/cm^2^ white light irradiation using HPF as a sensing probe. As shown in Figure S20A, the fluorescence intensity of HPF exhibited a gradual increase with prolonged irradiation time, demonstrating the ability of LIFM–ZY–3 to generate •OH under 20 mW/cm^2^ white light irradiation. Then, the •OH generation of the ligand L_3_ was examined under the same conditions in Figure S20B. The results revealed that ligand L_3_, at the same concentration as LIFM–ZY–3, could also generate •OH, though with significantly lower efficiency compared to the complex LIFM–ZY–3. And the fluorescence intensity of HPF had a negligible impact on the •OH generation compared to the complex LIFM–ZY–3, the ligand L_3_, and the HPF in Figure 2C and Figure S20C. Collectively, these findings demonstrated that the complex LIFM–ZY–3 could efficiently generate not only ^1^O_2_ but also •OH upon white light irradiation, confirming its potential as a versatile photosensitizer for type I PDT.

Additionally, we utilized the dihydroethidium (DHE) to detect the superoxide anions (O_2_^−^) [27]. As shown in Figure S21A, the fluorescence intensity of DHE gradually increased with prolonged irradiation time, demonstrating the ability of LIFM–ZY–3 to generate O_2_^−^ under 20 mW/cm^2^ white light irradiation. The O_2_^−^ production capability of the L_3_ was tested at the same concentration as LIFM–ZY–3 in Figure S21B. The results indicated that the O_2_^−^ generation efficiency of ligand L_3_ was only half that of the complex LIFM–ZY–3. As a control, we confirmed that DHE itself had no noticeable impact on the O_2_^−^ generation abilities of complex LIFM–ZY–3 and ligand L_3_ in Figure 2D and Figure S21C. These experimental results demonstrated that the complex LIFM–ZY–3 could efficiently generate a variety of ROS, including ^1^O_2_, •OH, and O_2_^−^, upon white light irradiation within a biologically tolerable range. The multifunctional ROS generation capability demonstrated the potential of LIFM–ZY–3 as a versatile PS for biological targeting and tumor therapy, emphasizing its significant promise for advanced biomedical applications.

**Figure 3:**
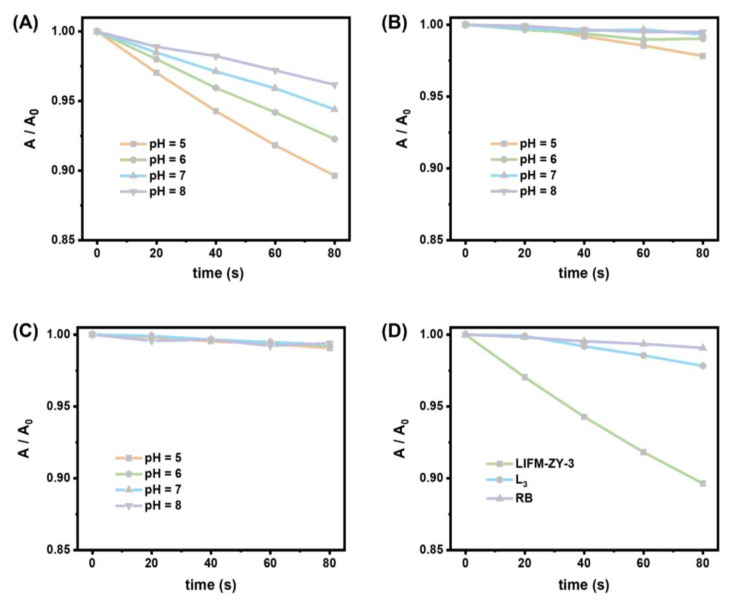
(**A**) The comparison diagram of the ability of the complex LIFM–ZY–3 to produce ^1^O_2_ by ABDA in PBS solutions with different pH (5, 6, 7, 8). (**B**) The comparison diagram of the ability of ligand L_3_ to produce ^1^O_2_ by ABDA in PBS solutions with different pH (5, 6, 7, 8). (**C**) A comparative diagram of the ability of dye RB to produce ^1^O_2_ by ABDA in PBS solutions with different pH (5, 6, 7, 8). (**D**) LIFM–ZY–3, L_3_, and RB produced ^1^O_2_ ability comparison diagrams by ABDA in pH = 5 PBS solution, respectively.

Considering the pH sensitivity of LIFM–ZY–3 in optical experiments, we investigated its ability to generate ^1^O_2_ under different pH conditions using ABDA under 20 mW/cm^2^ white light irradiation. The results revealed that the absorption of ABDA exhibited a gradual decrease with prolonged irradiation time, and the ^1^O_2_ generation efficiency of LIFM–ZY–3 was significantly enhanced at lower pH values, as illustrated in Figure 3A–C and Figures S22–S24. Compared to ligand L_3_ and RB, the complex LIFM–ZY–3 demonstrated significantly ^1^O_2_ production efficiency across a range of pH environments, as evidenced by the accelerated decay rate of ABDA in Figure 3D. And the ^1^O_2_ exhibited a characteristic near–infrared phosphorescence emission peak around 1270 nm, originating from its ¹Δg→³Σg^−^ transition. Based on this, we measured the spectrum of phosphorescence, it showed that the complex LIFM–ZY–3 had higher phosphorescent intensity with decreasing pH conditions in Figure S25. These results showed that LIFM–ZY–3 generated ^1^O_2_ more efficiently in acidic conditions, matching the acidic microenvironment of cancer cells, indicating that the complex LIFM–ZY–3 could enhance PDT efficacy in cancer cells compared to the neutral conditions of normal cells.

### 2.3. Subcellular Organelle Co-Localized Imaging

To further evaluate the biological suitability of LIFM–ZY–3, we initially assessed its cytotoxicity using the MTT assay. Given the PDT property of LIFM–ZY–3, we separately evaluated its phototoxicity and dark toxicity on HeLa cells. As shown in Figure S26, under dark conditions, the complex LIFM–ZY–3 exhibited minimal toxicity (95% cellular viability) to live cells; however, the cellular viability dropped to less than 40% under 20 mW/cm^2^ white light irradiation. It indicated that LIFM–ZY–3 could effectively exert its PDT effect in HeLa cells, making it a promising candidate for further in-depth studies in tumor therapy. Subsequently, we conducted cellular imaging at the subcellular organelle level in HeLa cells using the low-toxicity LIFM–ZY–3 under dark conditions. Then, we co–stained commercially available organelle targeting probes, including lysosome targeted deep red (LTDR), endoplasmic reticulum targeted red (ERTR), lipid droplet targeted red (LDTR), and mitochondria targeted deep red (MTDR), with the complex LIFM–ZY–3 in HeLa cells, as illustrated in Figure S27. The co-localization coefficient of LIFM–ZY–3 reached 0.89 when co–stained with LTDR, while the co-localization coefficients with other commercial dyes (MTDR, ERTR, and LDTR) were significantly lower at 0.41, 0.36, and 0.56, respectively. It demonstrated that the complex LIFM–ZY–3 exhibited subcellular organelle–targeting capabilities, with a specific ability to target lysosomes in HeLa cells.

### 2.4. Visualization of Lysosomal Viscosity Changes and Apoptosis

**Figure 4:**
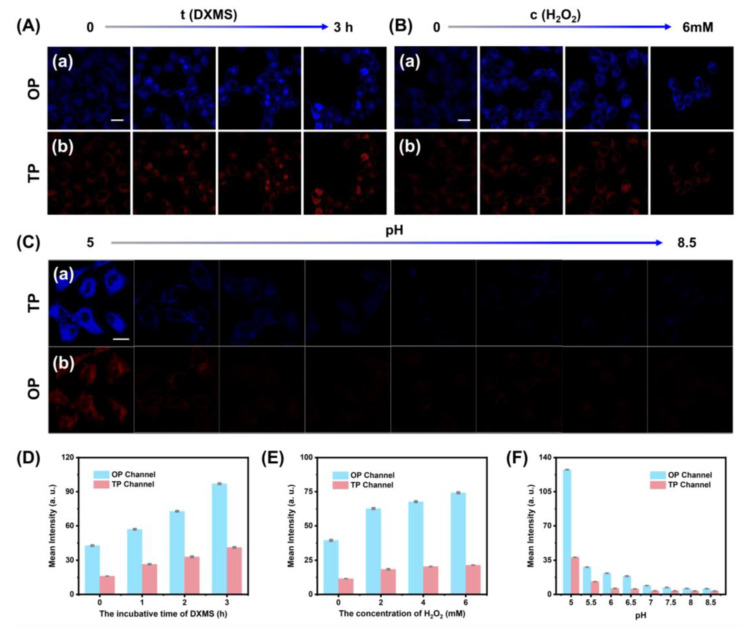
(**A**) HeLa cells were incubated with DXMS for 1 h, 2 h, and 3 h, respectively, and then the incubated HeLa cells were stained with LIFM–ZY–3 (5 μM): (a) OP confocal images of LIFM–ZY–3; (b) TP confocal images of LIFM–ZY–3. (**B**) HeLa cells were incubated with different concentrations of H_2_O_2_ (2 mM, 4 mM, 6 mM) for 0.5 h, and then the incubated HeLa cells were stained with LIFM–ZY–3 (5 μM) for 2 h: (a) OP confocal images of complex LIFM–ZY–3; (b) TP confocal images of LIFM–ZY–3. (**C**) HeLa cells were stained with LIFM–ZY–3 (5 μM) for 2 h, and then the incubated HeLa cells were incubated with different pH solutions of PBS (pH= 5.0, 5.5, 6.0, 6.5, 7.0, 7.5, 8.0, 8.5) for 15 min in OP channel (a) and TP channel (b). (**D**) The (**A**) mean intensity of LIFM–ZY–2. (**E**) The (**B**) mean intensity of LIFM–ZY–2. (**F**) The (**C**) mean intensity of LIFM–ZY–2. Scale bar = 20 μm.

According to previous literature, lysosomal viscosity could significantly increase following the stimulation of cells with dexamethasone (DXMS) [28]. HeLa cells were pretreated with dexamethasone (DXMS) for 1 h, 2 h, and 3 h, respectively, followed by incubation with LIFM–ZY–3 for 2 h. The complex LIFM–ZY–3 exhibited obvious fluorescence enhancement with increasing DXMS incubation time in both OP and TP channels in Figure 4A,D and Figure S28. It indicated that LIFM–ZY–3 could monitor viscosity changes induced by DXMS through fluorescence enhancement. According to related literature, an increase in cytoplasmic viscosity, often accompanied by lysosomal membrane permeabilization, serves as a key marker of apoptosis [29]. Hydrogen peroxide (H_2_O_2_) can induce cells to produce elevated levels of cytotoxic ROS, and high concentrations of ROS can cause oxidative damage in tumor cells, ultimately leading to apoptosis [30]. Based on this, HeLa cells were pretreated with varying concentrations of H_2_O_2_ (2 mM, 4 mM, 6 mM) for 0.5 h, followed by staining with LIFM–ZY–3 for 2 h. As shown in Figure 4B,E and Figure S29, LIFM–ZY–3 exhibited a clear fluorescence enhancement in both OP and TP channels with increasing H_2_O_2_ incubation concentration. Additionally, we induced apoptosis in cells using the pro–apoptotic drugs rotenone [31] and paclitaxel [32]. The experimental results demonstrated that LIFM–ZY–3 also exhibited significant fluorescence enhancement in HeLa cells following stimulation with pro–apoptotic drugs in Figure S30. It indicated that LIFM–ZY–3 could monitor H_2_O_2_-induced apoptosis through fluorescence enhancement, serving as evidence of its ability to self–report apoptosis following PDT treatment.

### 2.5. Visualization of Intracellular pH Changes

Based on the optical properties of LIFM–ZY–3, we conducted intracellular pH visualization experiments in HeLa cells. HeLa cells were incubated with LIFM–ZY–3 for 2 h, washed with PBS, treated with PBS solutions of different pH for 15 min, rinsed with PBS, and then subjected to confocal imaging. As shown in Figure 4C,F, LIFM–ZY–3 displayed gradually decreasing fluorescence intensity in both OP and TP channels with increasing pH conditions. Additionally, under strongly acidic conditions, the complex LIFM–ZY–3 lost the targeting lysosome specificity, suggesting that the strong acid disrupted the integrity of the subcellular organelles in HeLa cells, leading to their leakage into the cytoplasm and resulting in a rapid fluorescent enhancement. Unlike the acidic environment, the integrity of subcellular organelles in HeLa cells was disrupted under strongly alkaline conditions as well, achieving fluorescence quenching of LIFM–ZY–3. The differences in the above results indicated that LIFM–ZY–3 could have a highly sensitive response to low pH in HeLa cells, particularly in cancer cells, which were characterized by an acidic micro-environment. These findings demonstrated that LIFM–ZY–3 could monitor intracellular pH changes through fluorescence enhancement as acidity increased, highlighting its potential for intracellular pH self-reporting after PDT in solid tumors.

### 2.6. Photodynamic Therapy at the Cellular Level

**Figure 5:**
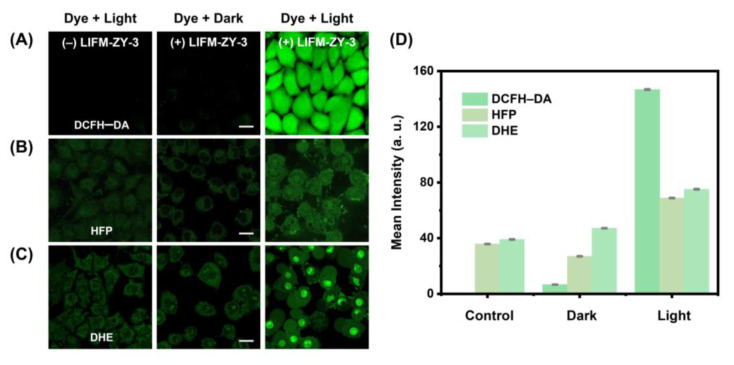
(**A**) HeLa cells were first incubated with LIFM–ZY–3 (5 μM) for 2 h, and then DCFH–DA was added for incubation for 20 min in both dark and light conditions to obtain confocal images. (**B**) HeLa cells were first incubated with LIFM–ZY–3 (5 μM) for 2 h, and then HFP was added for incubation for 20 min in both dark and light conditions to obtain confocal images. (**C**) HeLa cells were first incubated with LIFM–ZY–3 (5 μM) for 2 h, and then DHE was added for incubation for 20 min in both dark and light conditions to obtain confocal images. (**D**) The mean intensity of DCFH–DA, HFP, and DHE in both dark and light conditions. Scale bar = 20 μm.

Based on the ROS–related studies, the complex LIFM–ZY–3, compared to ligand L_3_ and RB, demonstrated the ability to generate multiple types of ROS (^1^O_2_, •OH, and O_2_^−^) under white light irradiation. These generated ROS could rapidly oxidize intracellular DNA, small molecules, and proteins, disrupting cellular functions and ultimately inducing apoptosis or necrosis in tumor cells [33]. Firstly, we employed the probe DCFH–DA to directly visualize and quantify the effective ROS levels in HeLa cells. As shown in Figure 5A,D, almost no green fluorescence of LIFM–ZY–3 was observed under the dark field condition in HeLa cells, indicating the absence of ROS generation without light irradiation. Also, there was no green fluorescence of DCFH–DA in the blank control group after light exposure, confirming the biologically safe light intensity. In contrast, widespread green fluorescence was observed in HeLa cells treated with LIFM–ZY–3 after light exposure, indicating that the complex LIFM–ZY–3 could generate ROS upon light irradiation in HeLa cells.

Then, we tested the ability of LIFM–ZY–3 to generate •OH using HPF at the cellular level in Figure 5B,D. The weak green fluorescence was observed in HeLa cells treated with LIFM–ZY–3 under dark conditions, suggesting the generation of a small amount of •OH even in the absence of light conditions. The fluorescence intensity in the blank control group without LIFM–ZY–3 under light condition matched that of cells incubated with LIFM–ZY–3 in the dark, indicating that the experimental light intensity induced an appropriate level of •OH production in blank HeLa cells. And a clear enhancement in fluorescence intensity was observed in HeLa cells treated with LIFM–ZY–3 under light condition, indicating that the complex LIFM–ZY–3 could generate •OH, consistent with the optical results.

In addition to the above studies, we also evaluated the ability of LIFM–ZY–3 to generate O_2_^−^ in HeLa cells using the commercial probe DHE. Under dark conditions, weak green fluorescence of LIFM–ZY–3 was observed in the cytoplasm of HeLa cells in Figure 5C,D, while DHE had not yet bound to the nucleus to form ethidium bromide, indicating minimal generation of O_2_^−^ in HeLa cells without light exposure. Under light conditions, HeLa cells without LIFM–ZY–3 incubation served as the blank control group, which showed negligible changes in fluorescence intensity but exhibited clear nuclear staining, demonstrating that the experimental light intensity induced an appropriate level of O_2_^−^ production in blank cells. It was evident that the fluorescence intensity and nuclear staining in HeLa cells treated with LIFM–ZY–3 were significantly enhanced compared to the blank control group under light conditions, manifesting that LIFM–ZY–3 could generate O_2_^−^ following light treatment in HeLa cells. All the above experiments demonstrated that the complex LIFM–ZY–3 possessed sufficient PDT capability to achieve the death of cancer cells, providing cellular-level data to support its potential for further application in tumor therapy and real-time monitoring.

## 3. Conclusions

In conclusion, we have successfully designed and synthesized LIFM–ZY–3, a TP fluorescent Zn(II) complex with lysosome-targeting properties. The complex not only exhibited dual responsiveness to pH and viscosity changes but also demonstrated excellent PDT efficacy against tumor cells. The lysosome-targeting capability of LIFM–ZY–3 was confirmed through cellular co-localization analysis. Using commercial probes as indicators, we verified that LIFM–ZY–3 generated multiple ROS, including ^1^O_2_, •OH, and O_2_^−^, thereby achieving the integration of Type I and II PDT under white light irradiation. This study highlighted that the fluorescent Zn(II) complexes could serve not only as potential PS but also achieve dual lysosomal pH and viscosity sensing, providing a structural foundation for the future design of ideal self-reporting fluorescent PS based on Zn(II) complexes. These findings underscored the potential of LIFM–ZY–3 as a versatile tool for tumor therapy and micro-environment monitoring, paving the way for tumor therapy.

## Data Availability

The original contributions presented in this study are included in the article. Further inquiries can be directed to the corresponding author.

## Supplementary Materials

The following supporting information can be downloaded at: https://www.mdpi.com/article/10.3390/molecules30112430/s1.
