# Supplementary material for: A Two-Photon Zn(II) Complex Photosensitizer with pH/Viscosity Dual Response for Enhanced Tumor Therapy
Source: Molecules. 2025 May 31;30(11):2430. doi: 10.3390/molecules30112430 (PMC12156442; doi:10.3390/molecules30112430)
Supplement: Supplementary file 1 [file molecules-30-02430-s001.zip › molecules-3618876-supplementary.pdf]

*Supporting Information for*

# A Two-Photon Zn(II) Complex Photosensitizer with pH/ Viscosity Dual Response for Enhanced Tumor Therapy

**Yu Zhang, Shao-Qi Guan, Ya-Ping Wang and Mei Pan ***

MOE Laboratory of Bioinorganic and Synthetic Chemistry, Lehn Institute of Functional Materials, IGCME, GBRCE for Functional Molecular Engineering, School of Chemistry, Sun Yat-sen University, Guangzhou 510006, China; zy6535337@163.com (Y.Z.); guanshq6@mail2.sysu.edu.cn (S.-Q.G.); yapingwang0393@163.com (Y.-P.W.)

* Correspondence: panm@mail.sysu.edu.cn

## Table of contents

**Pages**

Experimental Section ........ S3
Synthesis and characterization ........ S6
Scheme S1 ........ S7
Figure S1 ........ S8
Figure S2 ........ S8
Figure S3 ........ S8
Figure S4 ........ S9
Figure S5 ........ S9
Figure S6 ........ S9
Figure S7 ........ S10
Figure S8 ........ S10
Figure S9 ........ S10
Figure S10 ........ S11
Figure S11 ........ S11
Figure S12 ........ S12
Figure S13 ........ S12
Figure S14 ........ S12
Figure S15 ........ S13
Figure S16 ........ S13
Figure S17 ........ S13
Figure S18 ........ S14
Figure S19 ........ S14
Figure S20 ........ S14
Figure S21 ........ S15
Figure S22 ........ S15
Figure S23 ........ S16
Figure S24 ........ S17
Figure S25 ........ S17
Figure S26 ........ S18
Figure S27 ........ S18
Figure S28 ........ S19
Figure S29 ........ S19
Figure S30 ........ S20
Figure S31 ........ S20
Table S1 ........ S21

## Experimental Section

### Materials

All starting materials were purchased from commercial sources and used as received, and the solvents used for synthesis were of analytical grade, unless stated otherwise. For the biological section, Dulbecco's Modified Eagle’s Medium (DMEM, Life Technologies, USA), Penicillin/Streptomycin (Life Technologies, USA), 0.25% Trypsin-EDTA (Life Technologies, USA), Fetal Bovine Serum (FBS, Life Technologies, USA), Dulbecco’s Phosphate Buffered Saline (DPBS, Life Technologies, USA), 3-(4,5-dimethylthiazol-2-yl)-2,5-diphenyltetrazolium bromide (MTT, Sigma Aldrich, USA), Mito–Tracker Deep Red (Life Technologies, USA), LysoTracker Red (Beyotime, China), 2'-(4-ethoxyphenyl)-5-(4-methy-1-piperazinyl)-2,5'-bi-1H-benzimidazole trihydrochloride (Hoechst 33342, Beyotime, China) were used.

### Instrumentation

NMR spectra were recorded on a Bruker DPX 400 FT-NMR spectrometer 400 MHz or a Bruker DPX 500 (500 MHz) FT-NMR spectrometer with chemical shifts relative to tetramethylsilane (Me4Si). Electrospray ionization mass spectra (ESI-MS) were recorded on a Bruker Maxis 4G ESI–Q-TOF Bruker using electrospray ionization (positive-ion mode: ESI) in MeCN/water mixture solution. Electronic absorption spectra in the UV-visible region were recorded with a Shimadzu UV-3600 a Varian Cary 50 UV-vis spectrophotometer, in a 10×1 mm or 10×10 mm quartz cell. The photoluminescence spectra were measured on Edinburgh Instruments FLS 980 and FS 5. The cell viability was measured with a Tecan Infinite M200 Microplate Reader (Switzerland). All confocal images were taken with a Carl Zeiss LSM 880 laser scanning confocal microscope (Germany) combined with a Becker-Hickl time-correlated single photon counting system.

### General Procedures for Spectral Test

Unless special requirements, all reagents used in the spectrum testing were spectrally pure and applied without further purification. The water used in both the spectral and cell imaging experiments is ultrapure. The stock solution of the LIFM–ZY–3 (1 mM) was dissolved in dimethyl sulfoxide (DMSO) and stored at –20ºC. After thawing, it is used for spectral testing. Before the test, a solution of the LIFM–ZY–3 (5 μM, 3 mL) in different solvents was poured into a quartz absorption cell for the spectrum test. The excited wavelength of the probe was 360 nm and the emission ranged from 380 to 600 nm. Without special instructions, the concentration of LIFM–ZY–3 was 5 μM.

**Measurement of ROS generation rate**

The stock solution of LIFM–ZY–3 (1 mM) was prepared in dimethyl sulfoxide (DMSO) and stored at –20°C. After thawing, the solution was used for spectroscopic measurements. Prior to testing, LIFM–ZY–3 (5 μM, 3 mL) was dissolved in various solvents and transferred to a quartz cuvette for spectroscopic analysis (UV-visible absorption and fluorescence). Under irradiation with a 20 mW/cm² white light source, the production rates of $^1O_2$, •OH, and $O_2^-$ by LIFM–ZY–3 were determined using ABDA, HPF, and DHE, respectively.

**Cell culture**

HeLa cells were obtained from the Experimental Animal Center of Sun Yat-sen University. The cells were cultured in Dulbecco's Modified Eagle Medium (DMEM) supplemented with 10% fetal bovine serum (FBS) and 1% penicillin/streptomycin, and maintained at 37°C in a humidified atmosphere containing 5% $CO_2$. For optical measurements, the excitation and emission wavelengths were set as follows: OP mode: $\lambda_{ex}$= 405 nm, $\lambda_{em}$= 430–600 nm; TP mode: $\lambda_{ex}$= 690 nm, $\lambda_{em}$= 430–600 nm. The HeLa cells were cultured with LIFM–ZY–3 (5 μM) containing cell media (89% DMEM, 10% FBS, 1% double resistance) for 2 h.

**Cell imaging**

The cytotoxicity of the complex LIFM–ZY–3 at various concentrations was evaluated using the MTT assay. HeLa cells were seeded in a 96-well plate at a density of 5,000 cells per well and cultured for 12 h. Subsequently, LIFM–ZY–3 was diluted in DMSO at concentrations ranging from 1 to 10 μM. The samples were immediately diluted with fresh cell culture medium containing 1% (v/v) DMSO. Then, 20 μL of MTT solution (5 mg/mL) was added to each well, and the cells were further incubated for 4 h. After careful removal of the culture medium, 200 μL of DMSO was added to each well to dissolve the formazan crystals. The 96-well plate was gently shaken for 10 min, and the absorbance at 595 nm was recorded using a microplate reader. Cells treated with 1% (v/v) DMSO were used as the blank control group.

Prior to subcellular localization, HeLa cells were pretreated with LIFM–ZY–3 (5 μM) at 37°C for 2 h. Subsequently, the cells were co-stained with Mito Tracker Deep Red (MTDR, 150 nM, 15 min), Lyso Tracker Deep Red (LTDR, 150 nM, 30 min), ER Tracker Red (ERTR, 1 μM, 30 min), and LDs Tracker Red (LDTR, 0.5 μM, 30 min) at 37°C. After washing three times with PBS, the cells were imaged and recorded using a confocal microscope. The excitation wavelength for LIFM–

ZY–3 was set at 405 nm, while the excitation wavelengths for MTDR, LTDR, ERTR, and LDTR were 644 nm, 668 nm, 587 nm, and 552 nm, respectively. The emission wavelength ranges were as follows: 500 ± 50 nm (LIFM–ZY–3), 630 ± 20 nm (MTDR), 650 ± 20 nm (LTDR), 620 ± 20 nm (ERTR), and 580 ± 20 nm (LDTR).

HeLa cells were cultured in 35 mm confocal dishes for 24 h. Subsequently, the culture medium was removed, and the cells were treated with PBS buffer solutions at different pH values (5, 5.5, 6, 6.5, 7, 7.5, 8, 8.5), followed by the addition of cell culture medium containing LIFM–ZY–3 (5 μM). After washing three times with PBS, the HeLa cells were imaged using a confocal microscope.

HeLa cells were cultured in 35 mm confocal dishes for 24 h. Subsequently, the culture medium was removed, and the cells were treated with the viscosity-stimulating drug dexamethasone (DXMS, 5 μM) for 1 h, 2 h, and 3 h, respectively. Following this, the cells were incubated with culture medium containing LIFM–ZY–3 (5 μM). After washing three times with PBS, the cells were imaged using a confocal microscope.

HeLa cells were cultured in 35 mm confocal dishes for 24 h. Subsequently, the culture medium was removed, and the cells were treated with different concentrations of $H_2O_2$ (2 mM, 4 mM, 6 mM) for 30 minutes. Following this, the HeLa cells were incubated with culture medium containing LIFM–ZY–3 (5 μM). After washing three times with PBS, the cells were imaged using a confocal microscope.

The OP fluorescence signal of LIFM–ZY–3 was recorded in the blue fluorescence channel ($\lambda_{ex}$= 405 nm, $\lambda_{em}$= 430–600 nm), while the TP fluorescence signal was recorded in the red fluorescence channel ($\lambda_{ex}$= 690 nm, $\lambda_{em}$= 430–600 nm).

Without special instructions, the concentration of LIFM–ZY–3 was 5 μM.

**Intracellular ROS assay**

The ROS generation induced by LIFM–ZY–3: Firstly, HeLa cells were seeded in 35 mm confocal dishes and cultured for 24 h. Subsequently, the cells were incubated with culture medium containing LIFM–ZY–3 (5 μM) for 2 h. Following this, the cells were exposed to white light (20 mW/cm$^2$) for 30 min. Finally, the culture medium was removed, and the cells were incubated with 10 μM DCFH–DA for 20 minutes. After washing three times with PBS, the cells were imaged using a confocal microscope. The fluorescence signal of the ROS indicator DCFH–DA was recorded in the green fluorescence channel ($\lambda_{ex}$= 488 nm, $\lambda_{em}$= 510–600 nm).

The •OH generation induced by LIFM–ZY–3: Prior to confocal imaging, HeLa cells were cultured in 35 mm confocal dishes for 24 h. Firstly, the HeLa cells were incubated with culture medium containing LIFM–ZY–3 (5 μM) for 2 h. Subsequently, the cells were exposed to white light (20 mW/cm$^2$) for 30 min. Finally, the culture medium was removed, and the cells were incubated with 5 μM HPF for 30 minutes. After washing three times with PBS, the cells were imaged using a confocal microscope. The fluorescence signal of the •OH indicator HPF was recorded in the green fluorescence channel ($\lambda_{ex}$= 488 nm, $\lambda_{em}$= 510–600 nm).

The $O_2^{-}$: generation induced by LIFM–ZY–3: Prior to confocal imaging, HeLa cells were cultured in 35 mm confocal dishes for 24 h. Firstly, the HeLa cells were incubated with culture medium containing LIFM–ZY–3 (5 μM) for 2 h. Subsequently, the cells were exposed to white light (20 mW/cm$^2$) for 30 min. Finally, the culture medium was removed, and the cells were incubated with 20 μM DHE for 30 min. After washing three times with PBS, the cells were imaged using a confocal microscope. The fluorescence signal of the $O_2^{-}$ indicator DHE was recorded in the green fluorescence channel ($\lambda_{ex}$= 488 nm, $\lambda_{em}$= 510–600 nm).

## Synthesis and characterization

### Synthesis of NAP–ML–Br

4-Bromo-1,8-naphthalimide (10 mmol) was dissolved in dimethyl sulfoxide (DMSO), and N-(2-aminoethyl)morpholine (15 mmol) was added dropwise to the mixture. The reaction was heated at 100°C for 12 h and then allowed to stand overnight. After the addition of ultrapure water, a precipitate formed. The solid was collected by filtration, washed with hot ethanol, and dried to yield a pale-yellow solid with an 80% yield. $^1$H NMR (400 MHz, DMSO–$d_6$) $\delta$ 8.66–8.49 (m, 1H), 8.34 (d, $J$= 7.9 Hz, 1H), 8.23 (d, $J$= 7.9 Hz, 1H), 8.01 (dd, $J$= 8.4, 7.3 Hz, 1H), 4.18 (t, $J$= 7.0 Hz, 1H), 3.53 (t, $J$= 4.6 Hz, 2H), 2.57 (t, $J$= 7.0 Hz, 1H), 2.47 (d, $J$ = 4.7 Hz, 3H). $^{13}$C NMR (101 MHz, DMSO–$d_6$) $\delta$ 163.38 (d, $J$= 4.7 Hz), 133.18, 132.15, 131.91, 131.54, 130.34, 129.67, 129.36, 128.83, 123.24, 122.47, 66.64, 55.90, 53.84, 37.38.

### Synthesis of the ligand $L_3$

NAP–ML–Br (5 mmol) and 4'-(4-aminophenyl)-2,2':6',2''--terpyridine were separately dissolved in DMSO. Under a nitrogen atmosphere, the mixture was heated at 120°C for 12 h and then allowed to stand overnight. After the addition of ultrapure water, a precipitate formed. The solid was

collected by filtration, washed with hot ethanol, and dried to yield a pale–yellow solid with a 50% yield. $^{1}H$ NMR (400 MHz, DMSO–*d*) $\delta$ 8.86 (s, 1H), 8.72 (ddd, *J*= 24.2, 15.5, 6.3 Hz, 3H), 8.34 (d, *J*= 8.5 Hz, 1H), 8.12 (d, *J*= 7.8 Hz, 1H), 7.93 (t, *J*= 7.8 Hz, 1H), 7.84–7.73 (m, 1H), 7.69 (d, *J*= 7.8 Hz, 1H), 7.40 (t, *J*= 6.1 Hz, 1H), 4.41 (t, *J*= 6.9 Hz, 1H), 3.72 (t, *J*= 4.7 Hz, 2H), 2.77 (t, *J*= 7.0 Hz, 1H), 2.65 (s, 2H). $^{13}C$ NMR (101 MHz, DMSO–*d*) $\delta$ 164.23 (d, *J*= 20.6 Hz), 156.13 (d, *J*= 4.8 Hz), 149.44, 146.28, 139.13 (d, *J*= 62.8 Hz), 136.98, 132.60, 131.31, 130.87, 128.78, 127.80 (d, *J*= 22.6 Hz), 127.07, 124.01, 122.41 (d, *J*= 98.1 Hz), 118.87, 67.11, 56.20, 53.87, 37.30.

**Synthesis of the complex LIFM–ZY–3.**

$Zn(NO_3)_2\cdot 6H_2O$ (1 mmol), $L_3$ (1.2 mmol), and dimethylsulfoxide (DMSO, 9 mL, ultra-dry solvent) were combined in a 25 mL round-bottom flask and stirred at room temperature for 3 h. Ethyl acetate was then added to the mixture, resulting in the formation of a dark red precipitate. The precipitate was collected by centrifugation, washed, and dried to 90 mg yellowish powder, $ZnL_3$, 80.6% yield. $^{1}H$ NMR (400 MHz, DMSO–$d_6$) $\delta$ 9.54 (s, 1H), 9.21 (d, *J*= 8.1 Hz, 1H), 8.76–8.59 (m, 2H), 8.35 (q, *J* = 8.5, 8.1 Hz, 2H), 8.13–7.88 (m, 3H), 7.56 (t, *J*= 6.3 Hz, 1H), 4.27 (t, *J*= 6.8 Hz, 1H), 3.56 (t, *J*= 4.5 Hz, 2H), 2.65 (t, *J*= 7.0 Hz, 1H). $^{13}C$ NMR (101 MHz, DMSO–$d_6$) $\delta$ 198.38, 167.48, 160.27, 149.30, 140.65, 132.29, 130.33, 130.30, 130.14, 129.68, 128.21, 112.93, 108.85, 105.11, 49.88, 49.43, 49.07, 27.14, 21.58, 20.57, 20.04. The MS (ESI) m/z for LIFM–ZY–3 $\{[(C_{39}H_{31}N_5O_3Zn)^{2+}(OH)^{-}]^{+}+4H^{+}\}$: calcd: 638.2762, found: 638.1492.

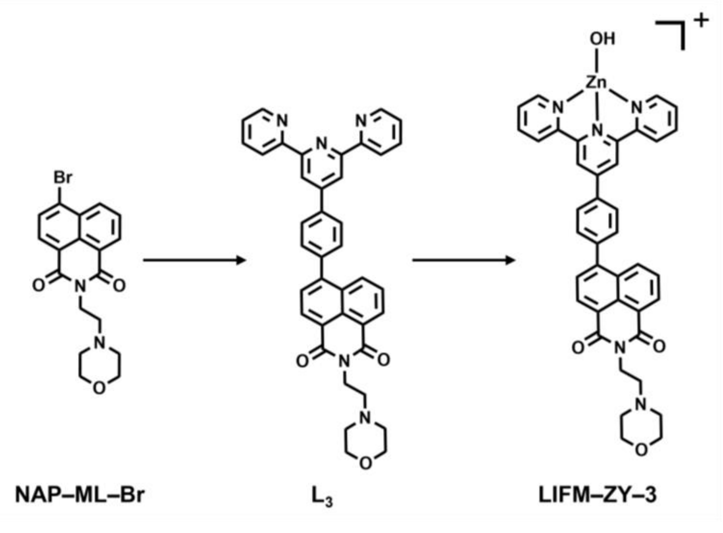


**Scheme S1** The synthetic routines of LIFM–ZY–3.

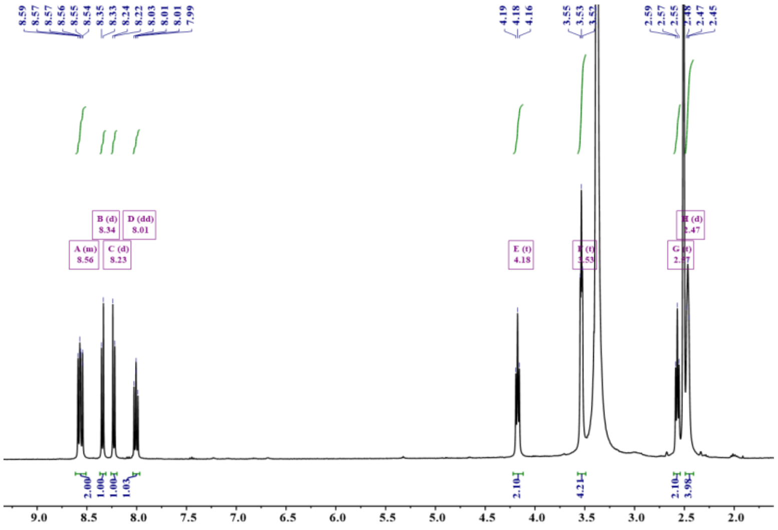


**Figure S1** $^{1}$H NMR spectrum of NAP–ML–Br in DMSO–$d_6$.

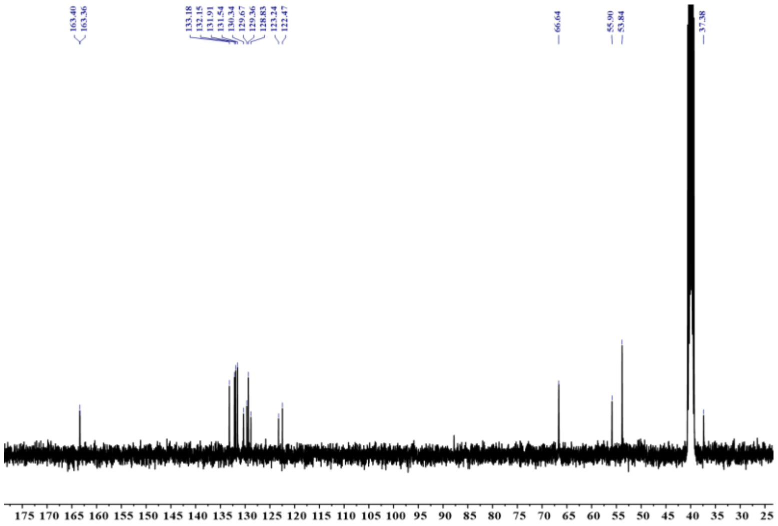


**Figure S2** $^{13}$C NMR spectrum of NAP–ML–Br in DMSO–$d_6$.

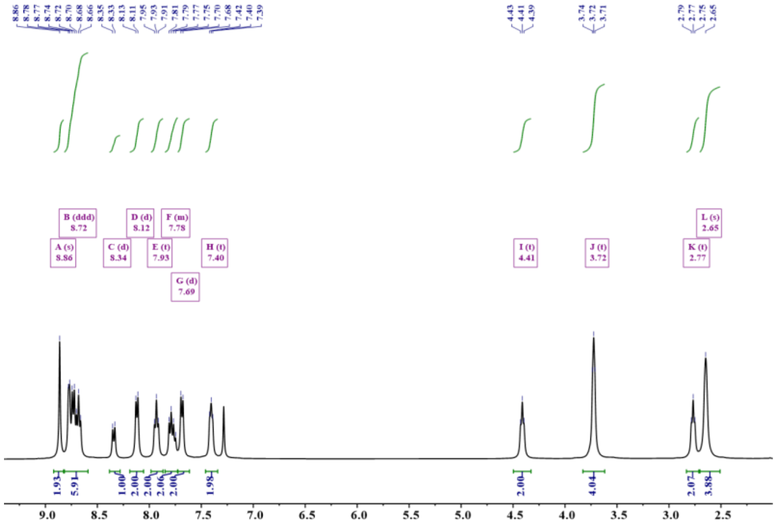


**Figure S3** $^{1}$H NMR spectrum of $L_3$ in DMSO–$d_6$.

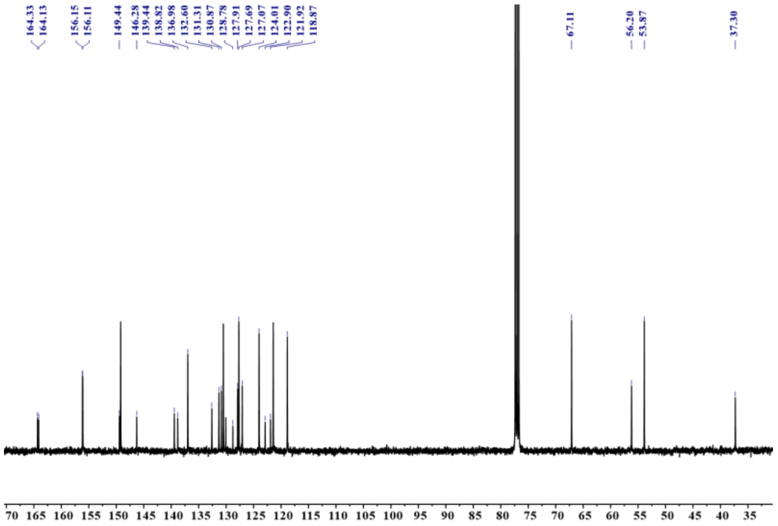

**Figure S4** $^{13}C$ NMR spectrum of $L_3$ in DMSO–$d_6$.

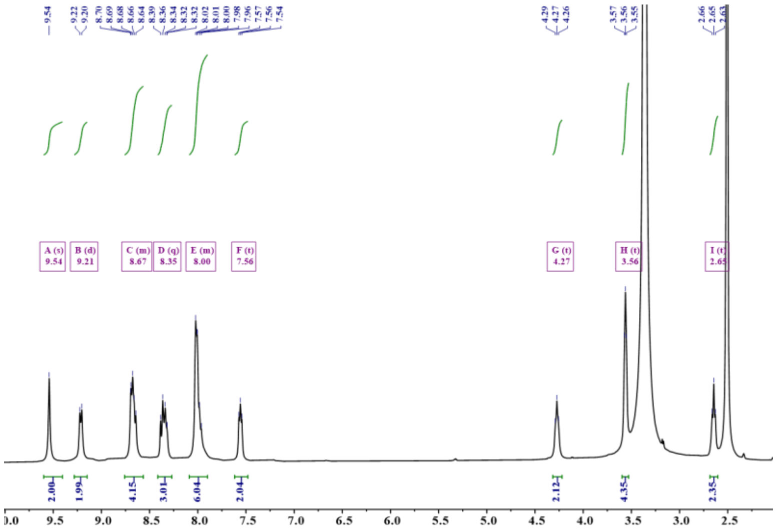

**Figure S5** $^{1}H$ NMR spectrum of LIFM–ZY–3 in DMSO–$d_6$.

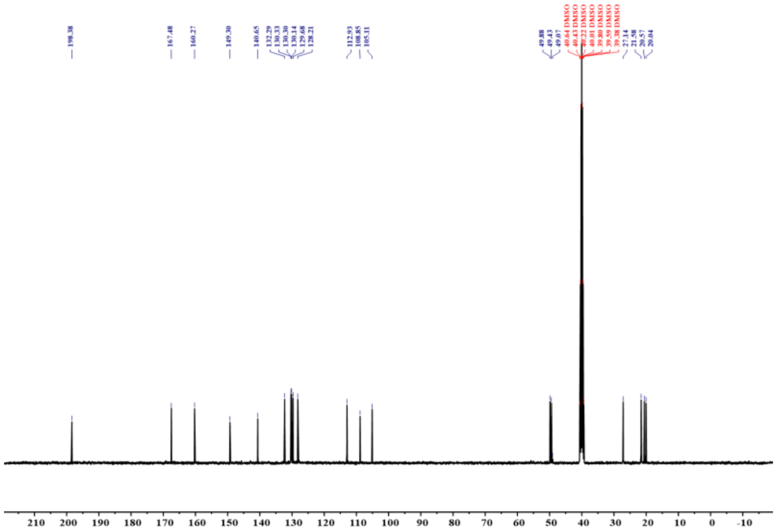

**Figure S6** $^{13}C$ NMR spectrum of LIFM–ZY–3 in DMSO–$d_6$.

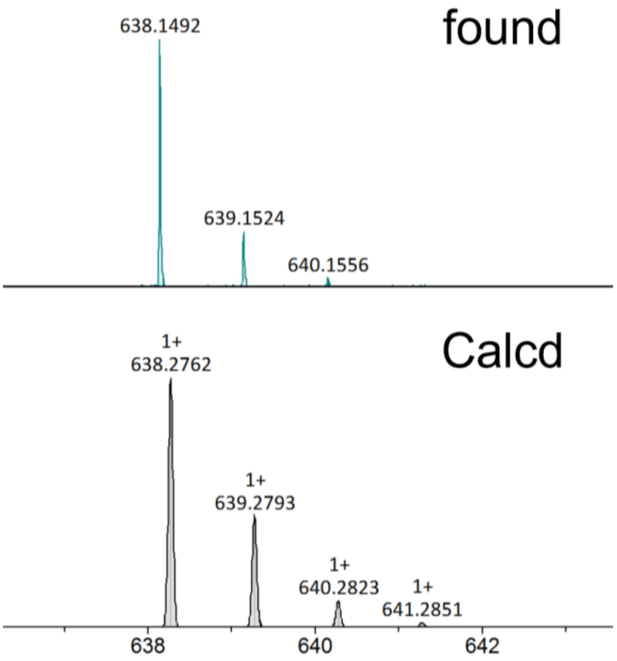


**Figure S7** The ESI–MS spectrum of LIFM–ZY–3.

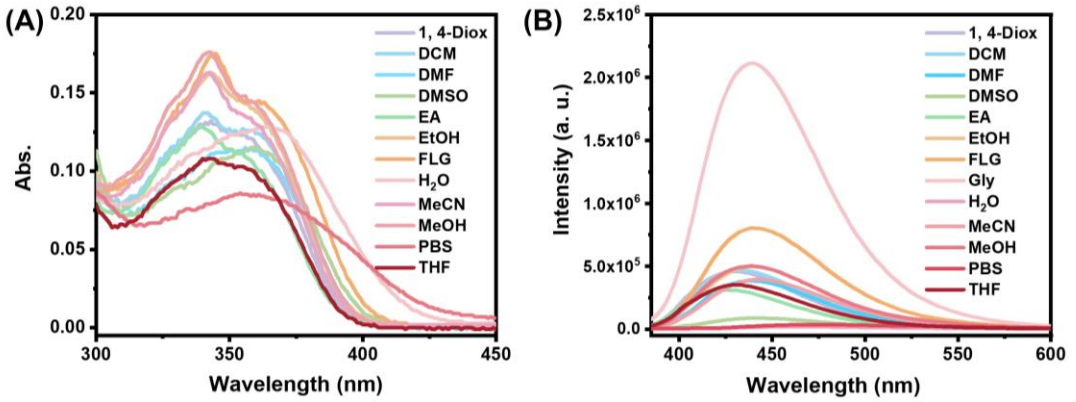


**Figure S8** (A) Absorption spectra of LIFM–ZY–3 (5 µM) in different solvents. (B) Emission spectra of LIFM–ZY–3 (5 µM) in different solvents.

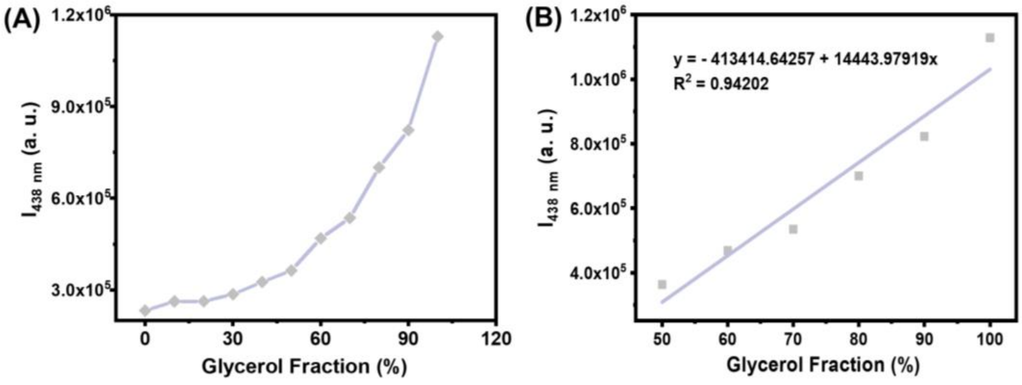


**Figure S9** (A) The line chart of the maximum fluorescence intensity of LIFM–ZY–3 (5 µM) at 438

nm versus different glycerol fraction of viscosity in EtOH/glycerol (v/v) mixtures. (B) The linear relationship of the emission maximum of LIFM–ZY–3 (5 μM) versus the fraction of glycerol in EtOH/glycerol (v/v) mixtures.

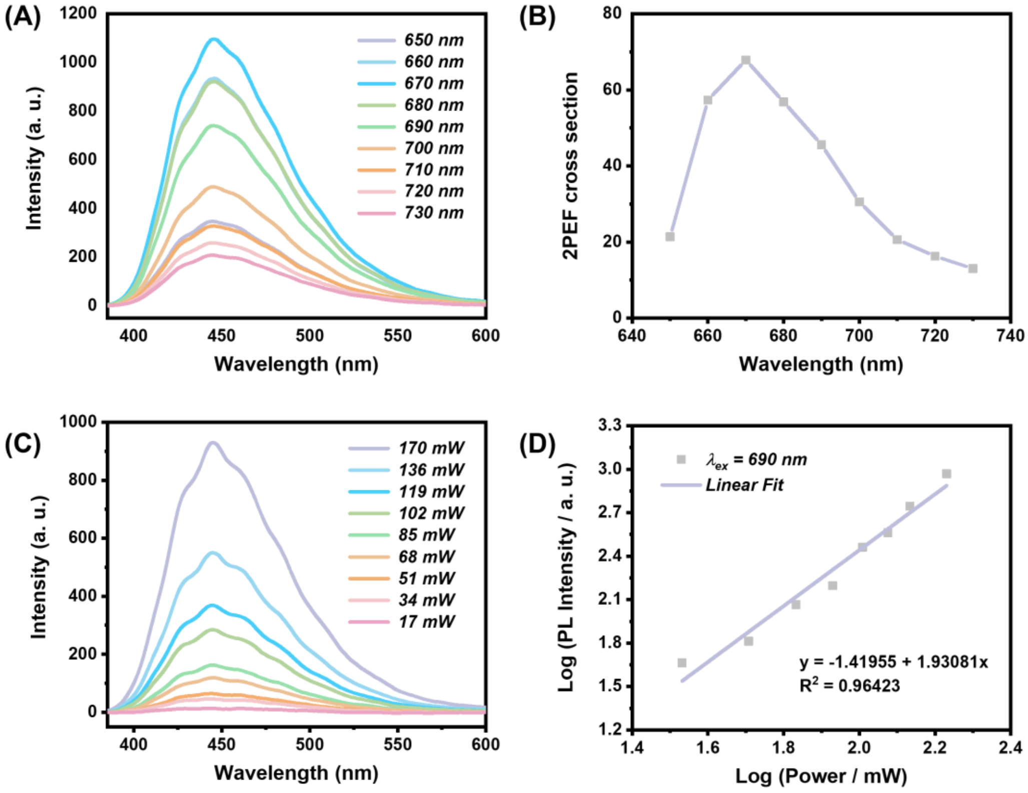


**Figure S10** (A) The TP emission spectra of LIFM–ZY–3 (10 μM) in DMSO. (B) The TP fluorescence cross section of LIFM–ZY–3 (10 μM) in DMSO. (C) TP emission spectra of LIFM–ZY–3 (10 μM). (D) The linear relationship of slope 2.

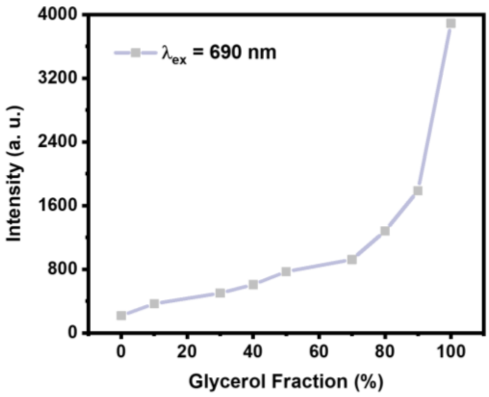


**Figure S11** The line chart of the maximum TP fluorescence intensity of LIFM–ZY–3 (10 μM) at 690 nm versus the changed viscosity media of the EtOH/glycerol (v/v) mixtures.

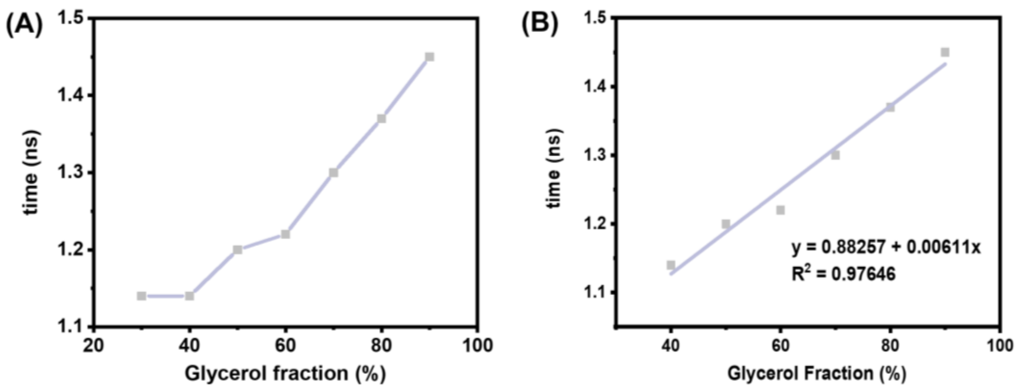


**Figure S12** (A) The line chart of the fluorescence lifetime of LIFM–ZY–3 (10 μM) versus the diverse glycerol fraction of viscosity in EtOH/glycerol (v/v) mixtures. (B) The linear relationship of fluorescence lifetime of LIFM–ZY–2 versus the diverse fraction of glycerol in water/glycerol (v/v) systems.

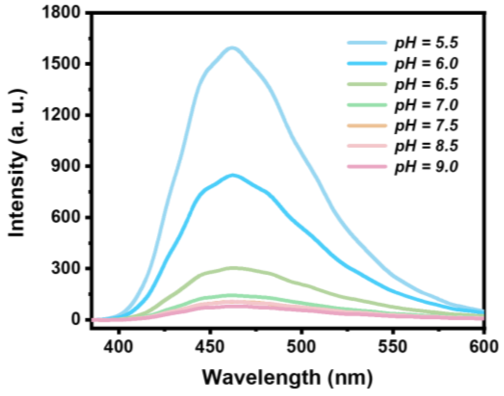


**Figure S13** TP fluorescence spectra of LIFM–ZY–3 (10 μM) in various pH conditions.

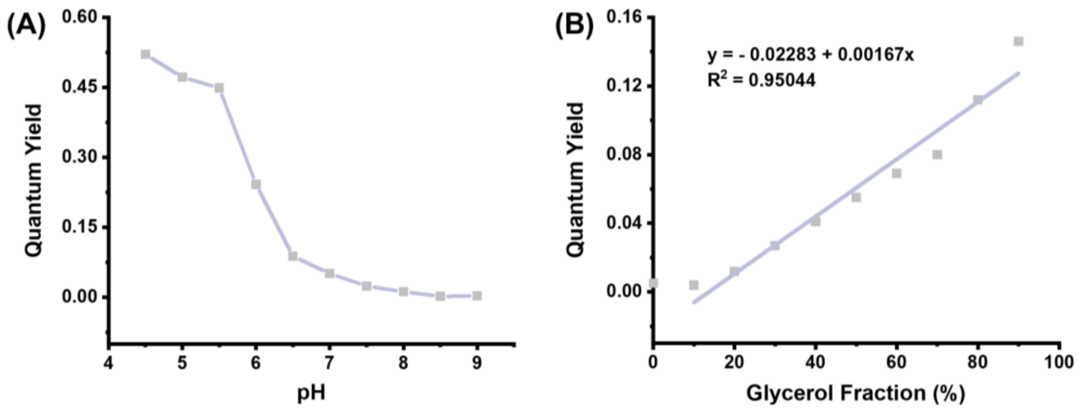


**Figure S14** (A) The fluorescence quantum yield of LIFM–ZY–3 (5 μM) in different pH conditions. (B) The linear relationship of LIFM–ZY–3 (5 μM) between the QY and the different glycerol fraction.

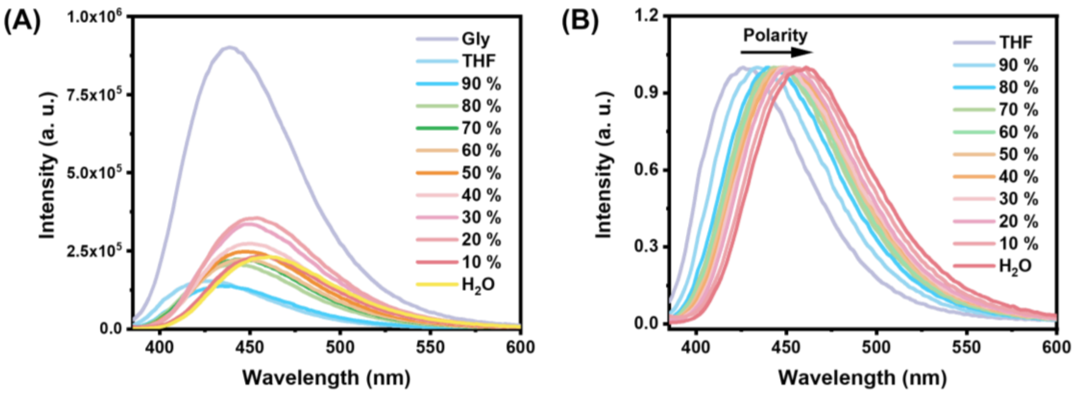


**Figure S15** (A) The fluorescence spectra of complex LIFM–ZY–3 (5 μM) in different polar systems (THF/$H_2O$) and glycerol. (B) Fluorescence spectra of LIFM–ZY–3 (5 μM) normalized in different polar systems (THF/$H_2O$).

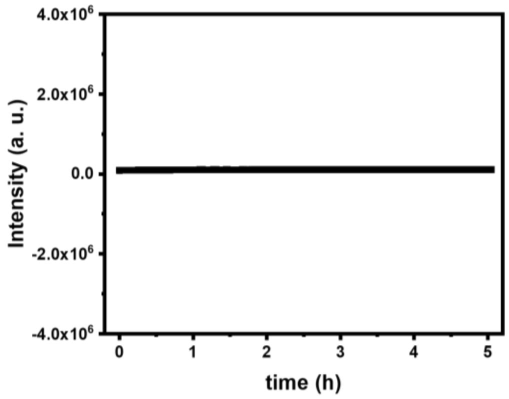


**Figure S16** The photostability test of LIFM–ZY–3 (5 μM) under continuous illumination for 8 h.

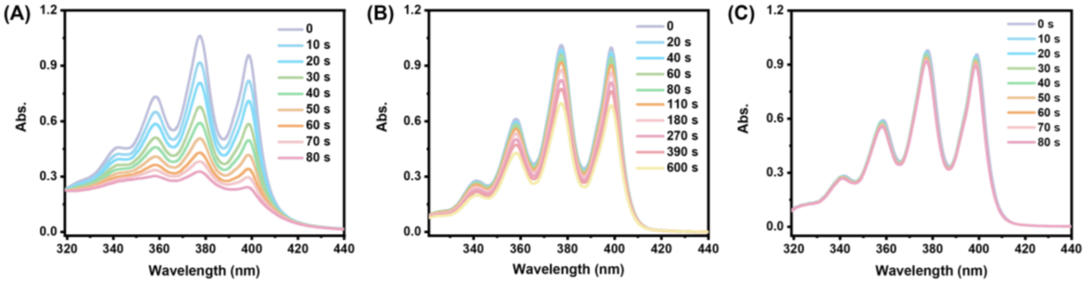


**Figure S17** (A) ABDA was used to monitor the ability of the complex LIFM–ZY–3 (20 μM) to produce $^1O_2$. (B) ABDA was used to monitor the ability of ligand $L_3$ (20 μM) to produce $^1O_2$. (C) ABDA was used to monitor the ability of dye RB (20 μM) to produce $^1O_2$, and the light source was 35 mW/cm$^2$ white light irradiation.

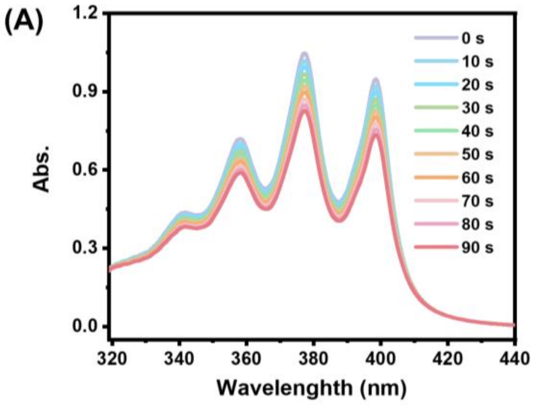


**Figure S18** (A) ABDA was used to monitor the ability of the complex LIFM–ZY–3 (20 μM) to produce $^1O_2$, and the light source was 20 mW/cm$^2$ white light irradiation. (B) The linear relationship between the absorption of LIFM–ZY–3 at 380 nm and the white light irradiation time.

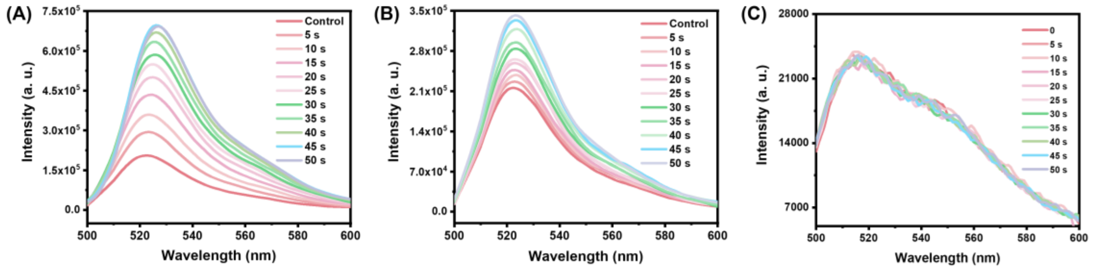


**Figure S19** (A) DCFH–DA was used to monitor the ability of the complex LIFM–ZY–3 (20 μM) to produce $^1O_2$. (B) DCFH–DA was used to monitor the ability of ligand $L_3$ (20 μM) to produce $^1O_2$. (C) The ability of DCFH–DA to produce $^1O_2$ under the irradiation of the same light source 20 mW/cm$^2$ white light irradiation.

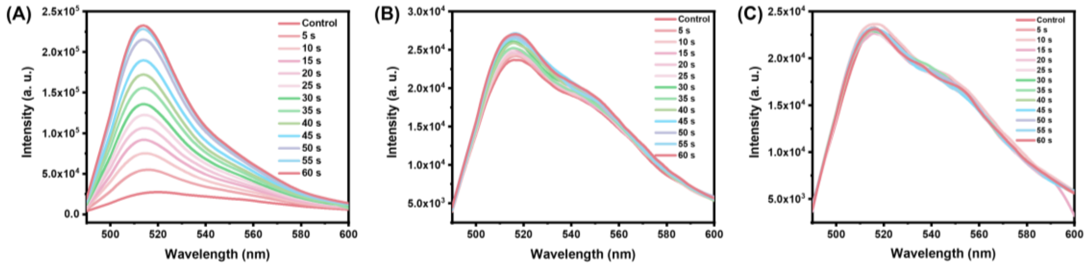


**Figure S20** (A) HPF was used to monitor the ability of the complex LIFM–ZY–3 (20 μM) to produce •OH. (B) HPF was used to monitor the ability of ligand $L_3$ (20 μM) to produce •OH. (C) The ability of HPF to produce •OH under 20 mW/cm$^2$ white light irradiation.

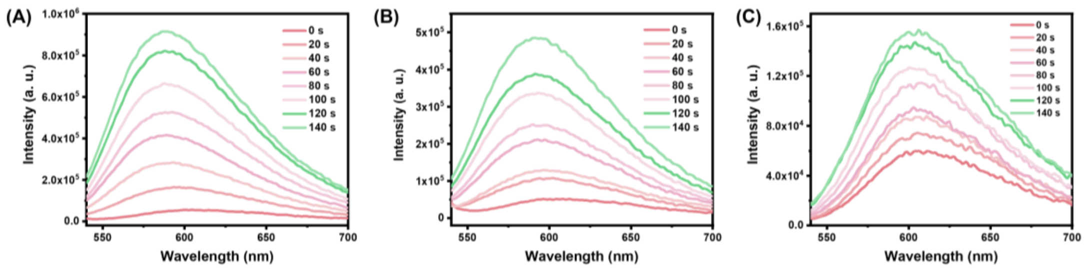


**Figure S21** (A) DHE was used to monitor the ability of the complex LIFM–ZY–3 (20 μM) to produce $O^{2-}$. (B) DHE was used to monitor the ability of ligand $L_3$ (20 μM) to produce $O^{2-}$. (C) The ability of HPF to produce $\bullet O^{2-}$ under 20 mW/cm$^2$ white light irradiation.

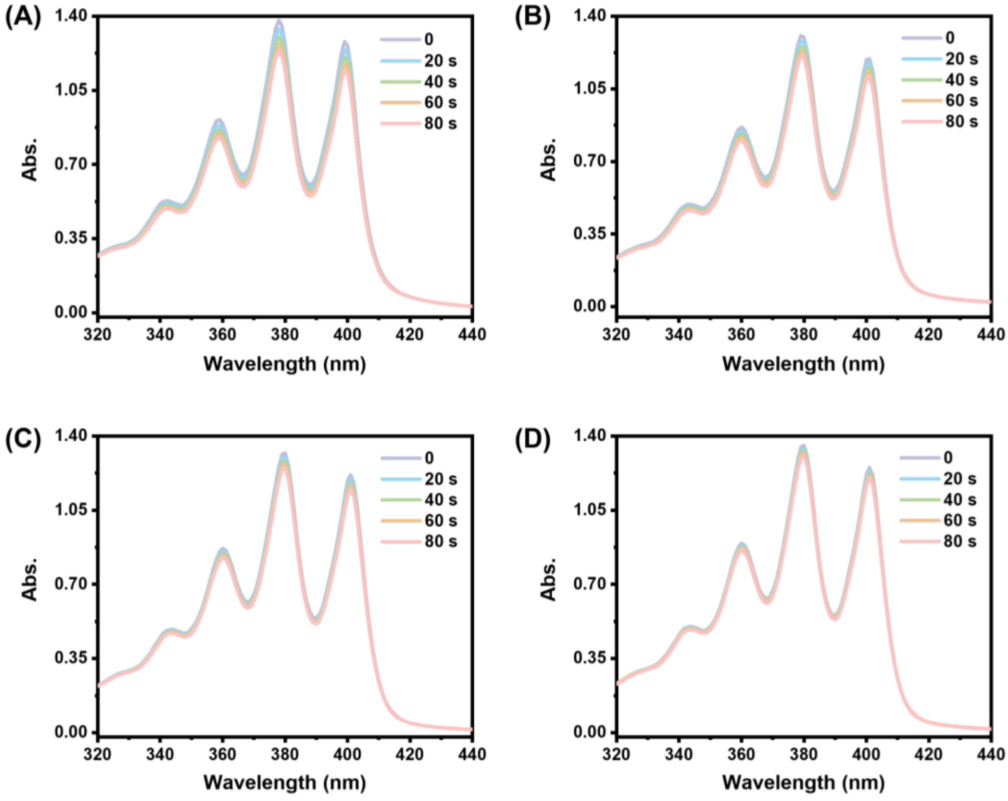


**Figure S22** (A) The ability of complex LIFM–ZY–3 (20 μM) to produce $^{1}O_2$ was monitored by ABDA in PBS solution at pH= 5. (B) ABDA was used to monitor the ability of LIFM–ZY–3 (20 μM) to produce $^{1}O_2$ in PBS solution at pH= 6. (C) The ability of LIFM–ZY–3 (20 μM) to produce $^{1}O_2$ was monitored by ABDA in PBS solution at pH= 7. (D) The ability of LIFM–ZY–3 (20 μM) to produce $^{1}O_2$ was monitored by ABDA in PBS solution with pH= 8, and the light source conditions were consistent at 20 mW/cm$^2$.

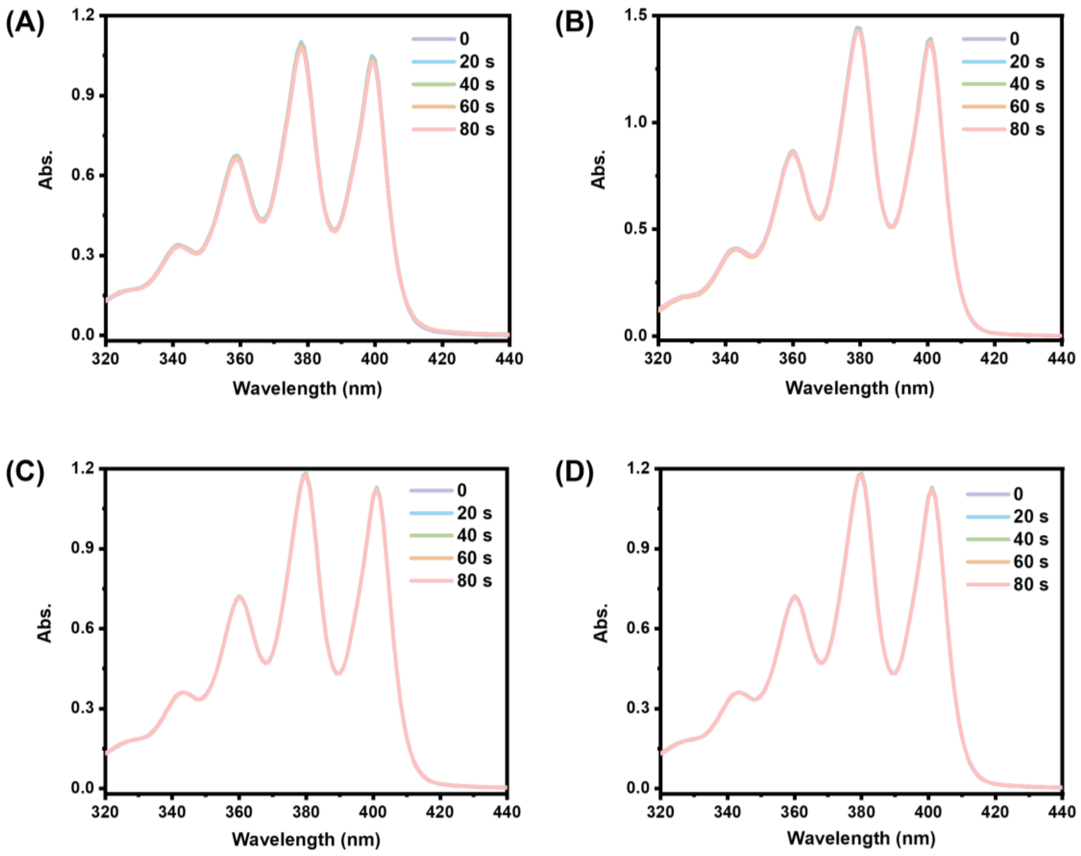


**Figure S23** (A) The ability of ligand $L_3$ (20 μM) to produce $^1O_2$ was monitored by ABDA in PBS solution at pH= 5. (B) ABDA was used to monitor the ability of $L_3$ (20 μM) to produce $^1O_2$ in PBS solution at pH= 6. (C) The ability of $L_3$ (20 μM) to produce $^1O_2$ was monitored by ABDA in PBS solution at pH= 7. (D) The ability of $L_3$ (20 μM) to produce $^1O_2$ was monitored by ABDA in PBS solution with pH= 8, and the light source conditions were consistent at 20 mW/cm$^2$.

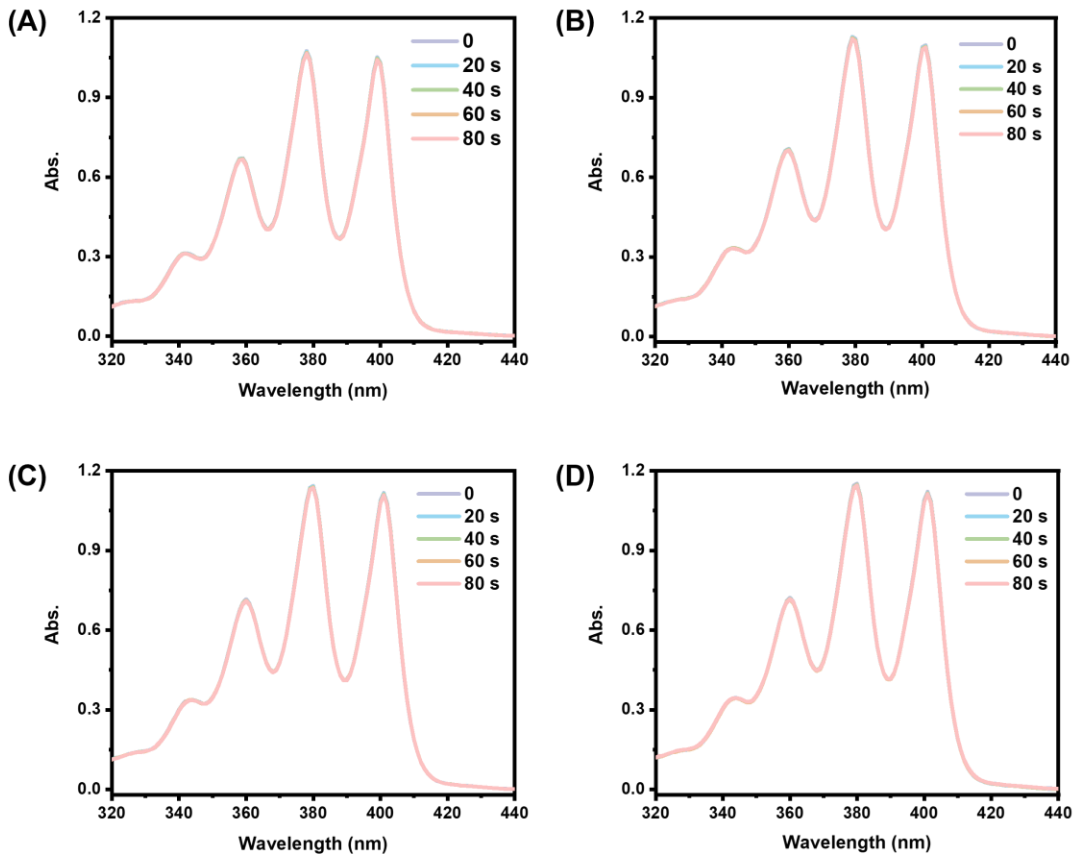


**Figure S24** (A) The ability of complex RB (20 μM) to produce $^1O_2$ was monitored by ABDA in PBS solution at pH= 5. (B) ABDA was used to monitor the ability of RB (20 μM) to produce $^1O_2$ in PBS solution at pH= 6. (C) The ability of RB (20 μM) to produce $^1O_2$ was monitored by ABDA in PBS solution at pH= 7. (D) The ability of RB (20 μM) to produce $^1O_2$ was monitored by ABDA in PBS solution with pH= 8, and the light source conditions were consistent at 20 mW/cm$^2$.

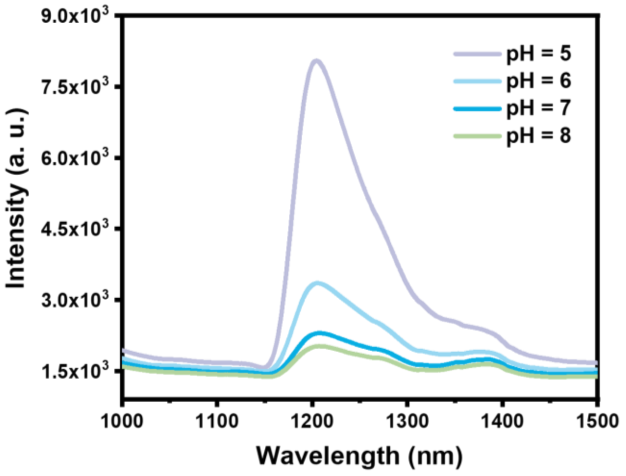


**Figure S25** The phosphorescent spectrums of LIFM–ZY–3 for generating $^1O_2$ in different pH conditions, and the light source conditions were consistent at 20 mW/cm$^2$.

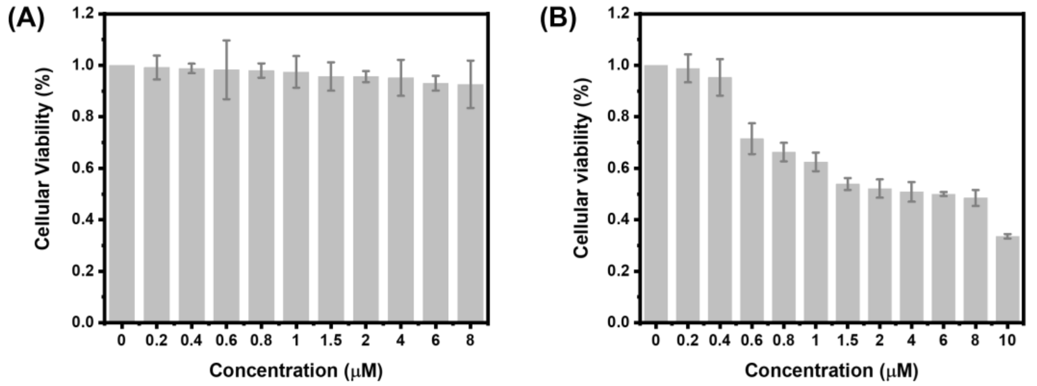


**Figure S26** (A) The MTT toxicity experiments of LIFM–ZY–3 under different concentrations (0–8 μM) for HeLa cells in dark environment. (B) The MTT toxicity experiments of LIFM–ZY–3 under different concentrations (0–8 μM) for HeLa cells which were irradiated with 20 mW/cm$^2$ white light for 30 min.

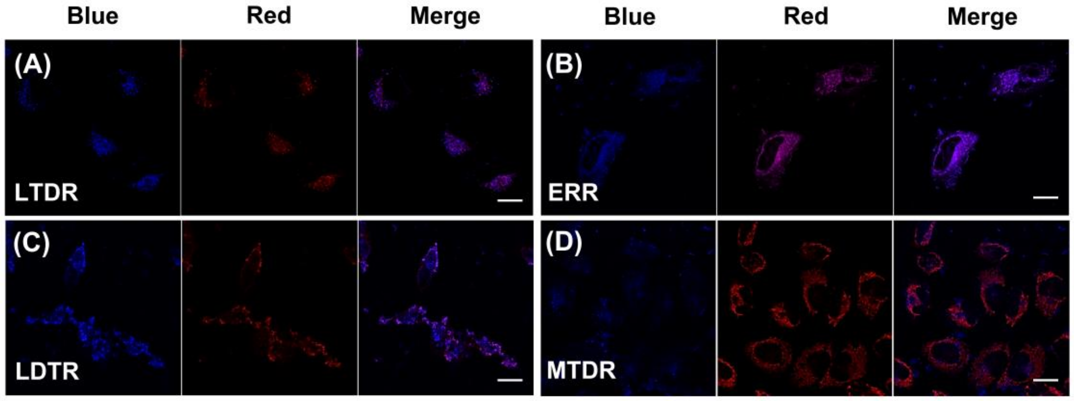


**Figure S27** The co–localization experiment of the complex LIFM–ZY–3 (5 μM) and different commercial organelle targeting dyes in HeLa cells: (A) LIFM–ZY–3, LTDR; (B) LIFM–ZY–3, MTDR; (C) LIFM–ZY–3, ETR; (D) LIFM–ZY–3, LTDR.

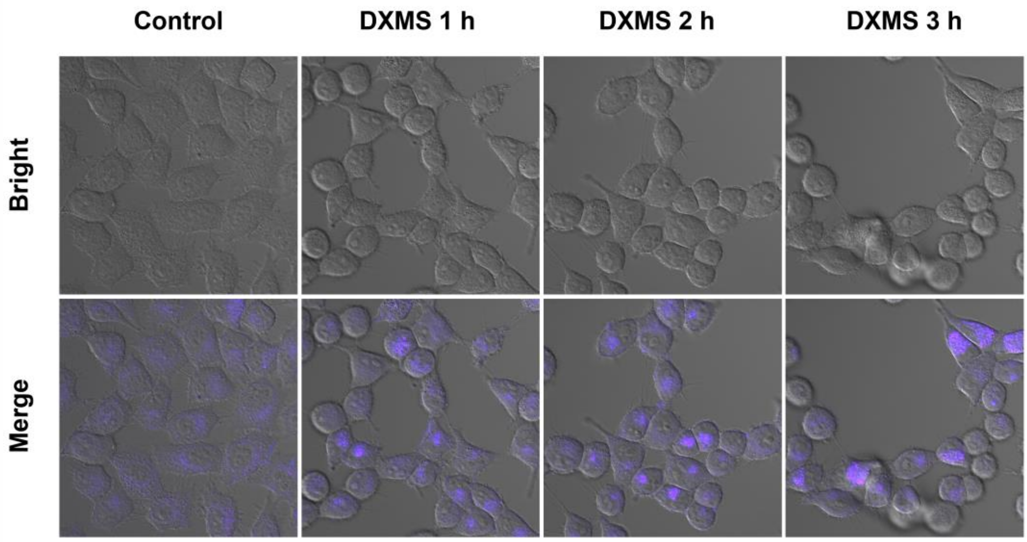


**Figure S28** The bright and merge channels of LIFM–ZY–3 that HeLa cells were incubated with DXMS for 1 h, 2 h, and 3 h, corresponding to the Figure 4A.

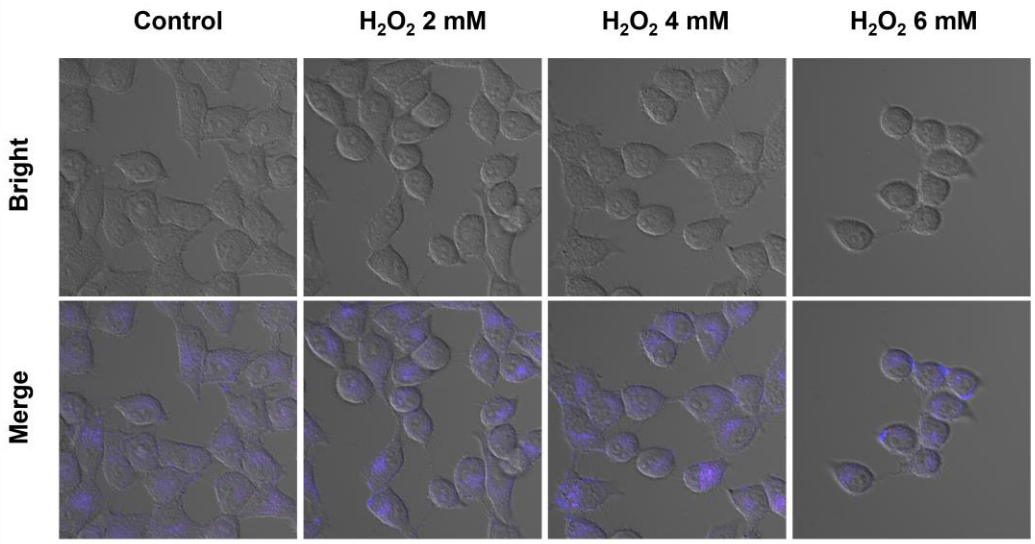


**Figure S29** The bright and merge channels of LIFM–ZY–3 that HeLa cells were incubated with different concentrations of $H_2O_2$ (2 mM, 4 mM, 6 mM) for 0.5 h, corresponding to the Figure 4B.

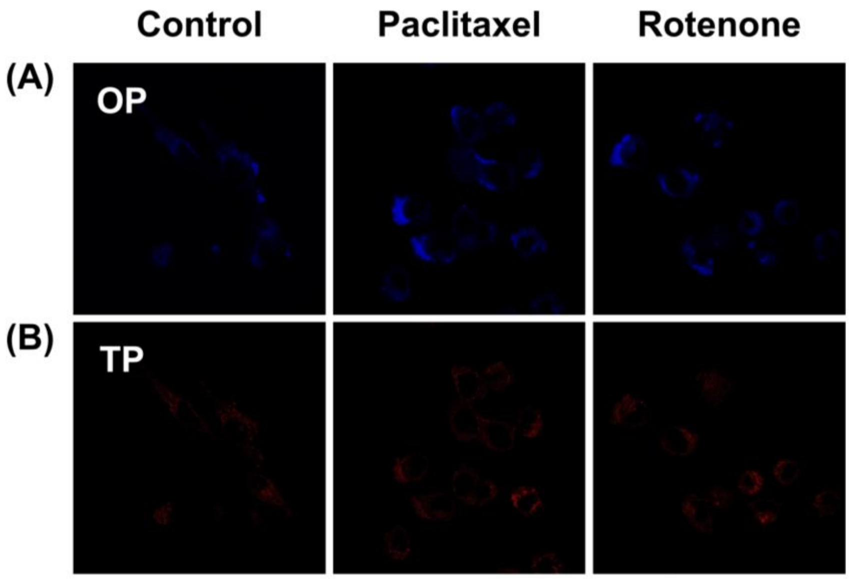


**Figure S30** HeLa cells were incubated with different pro–apoptotic drugs Paclitaxel (5μM) and Rotenone (20 μM) for 2 h, and then the incubated HeLa cells were stained with LIFM–ZY–3 (5 μM) for 2 h: (A) OP channel; (B) TP channel.

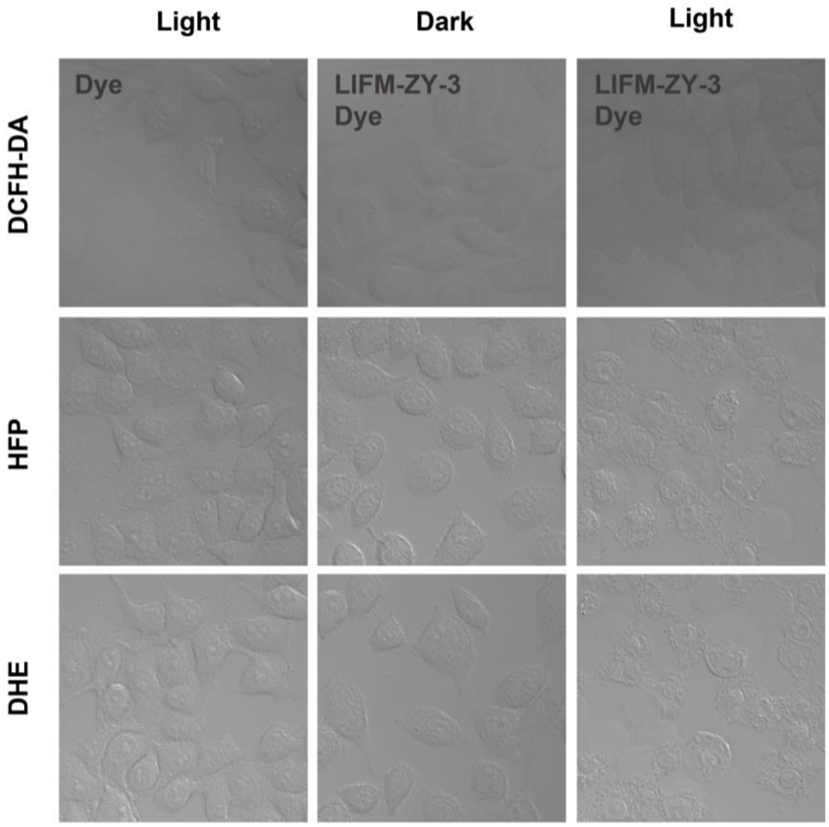


**Figure S31** The bright channel of LIFM–ZY–3 (5 μM) that HeLa cells were incubated with DCFH–DA (10 μM), HPF (5 μM), and DHE (20 μM) for 20 min, corresponding to the Figure 5A.

**Table S1** The maximum absorbance and emission wavelength and quantum yield of $L_2$ and LIFM–ZY–3 in diverse solvents.

| | $L_2$ | | LIFM–ZY–3 | |
|---|---|---|---|---|
| | $\lambda_{ex}$ / $\lambda_{em}$ nm | QY % | $\lambda_{ex}$ / $\lambda_{em}$ nm | QY % |
| **DCM** | 357 / 432 | 2.8 | 358 / 433 | 3.7 |
| **DMF** | 360 / 439 | 3.7 | 357 / 439 | 2.2 |
| **DMSO** | 359 / 438 | 2.3 | 359 / 439 | 9.6 |
| **EA** | 355 / 425 | 7.1 | 356 / 419 | 3.9 |
| **EtOH** | 352 / 443 | 7.5 | 355 / 437 | 7.6 |
| **FLG** | 344 / 442 | 11.6 | 362 / 440 | 15.3 |
| **$H_2O$** | 346 / 410 | 3.4 | 367 / 453 | 27.3 |
| **MeCN** | 342 / 438 | 4.1 | 359 / 438 | 0.9 |
| **MeOH** | 341 / 457 | 4.9 | 359 / 437 | 5.6 |
| **PBS** | 335 / 410 | 2.3 | 370 / 470 | 28.4 |
| **THF** | 352 / 424 | 3.4 | 356 / 430 | 4.2 |
| **1, 4-Diox** | 355 / 424 | 2.3 | 355 / 427 | 0.6 |
| **$^1O_2$ generation** | --- | 0.69 | --- | 7.82 |
| | The $^1O_2$ quantum yield (Φ) is calculated by $\Phi_\Delta=\frac{\Delta A_{sample}}{\Delta A_{RhB}}\bullet\Phi_{RhB}$, the $^1O_2$ quantum yield of RhB was 0.57 as a reference. | | | |
